# Convergent evolution of a mobile bony tongue in flighted dinosaurs and pterosaurs

**DOI:** 10.1371/journal.pone.0198078

**Published:** 2018-06-20

**Authors:** Zhiheng Li, Zhonghe Zhou, Julia A. Clarke

**Affiliations:** 1 Key Laboratory of Vertebrate Evolution and Human Origins of Chinese Academy of Sciences, Institute of Vertebrate Paleontology and Paleoanthropology, Chinese Academy of Sciences, Beijing, China; 2 CAS Center for Excellence in Life and Paleoenvironment, Beijing, China; 3 Department of Geological Sciences, University of Texas at Austin, Austin, Texas, United States of America; University of Michigan, UNITED STATES

## Abstract

The tongue, with fleshy, muscular, and bony components, is an innovation of the earliest land-dwelling vertebrates with key functions in both feeding and respiration. Here, we bring together evidence from preserved hyoid elements from dinosaurs and outgroup archosaurs, including pterosaurs, with enhanced contrast x-ray computed tomography data from extant taxa. Midline ossification is a key component of the origin of an avian hyoid. The elaboration of the avian tongue includes the evolution of multiple novel midline hyoid bones and a larynx suspended caudal to these midline elements. While variable in dentition and skull shape, most bird-line archosaurs show a simple hyoid structure. Bony, or well-mineralized, hyoid structures in dinosaurs show limited modification in response to dietary shifts and across significant changes in body-size. In Dinosauria, at least one such narrow, midline element is variably mineralized in some basal paravian theropods. Only in derived ornithischians, pterosaurs and birds is further significant hyoid elaboration recorded. Furthermore, only in the latter two taxa does the bony tongue structure include elongation of paired hyobranchial elements that have been associated in functional studies with hyolingual mobility. Pterosaurs and enantiornithine birds achieve similar elongation and inferred mobility via elongation of ceratobranchial elements while within ornithurine birds, including living Aves, ossified and separate paired epibranchial elements (caudal to the ceratobranchials) confer an increase in hyobranchial length. The mobile tongues seen in living birds may be present in other flighted archosaurs showing a similar elongation. Shifts from hypercarnivory to more diverse feeding ecologies and diets, with the evolution of novel locomotor strategies like flight, may explain the evolution of more complex tongue function.

## Introduction

**Fig 1:**
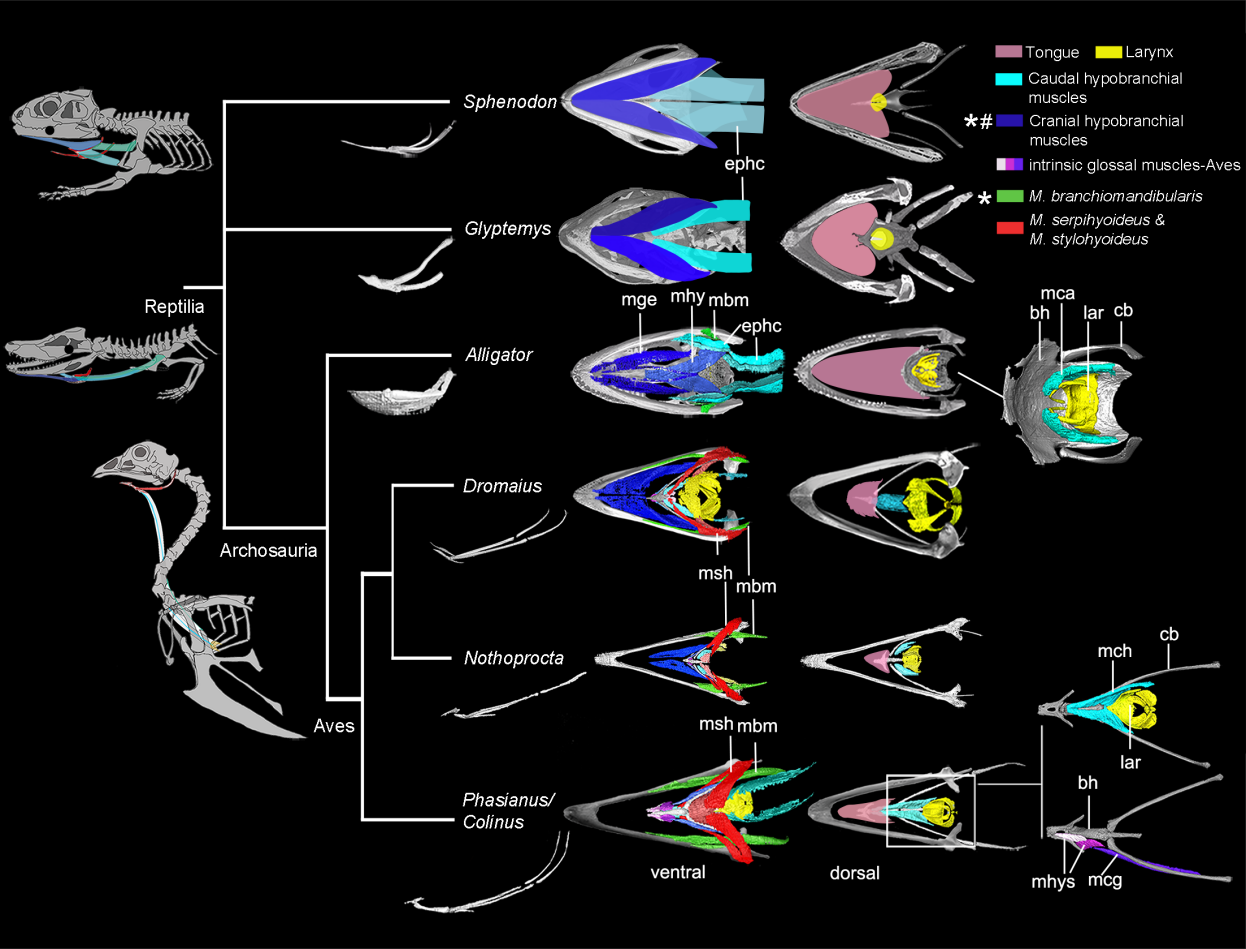
Muscular, fleshy, bone or cartilage elements of the tongue in extant archosaurs and outgroups.

The tongue of terrestrial vertebrates differs markedly related to divergence in feeding and respiratory strategies [1, 2]. Within extant Archosauria, birds display diverse tongue morphologies associated with radiation in feeding ecologies [1]. In Aves, extensive ossification of tongue elements occurs not only in the elongated paired ceratobranchials and epibranchials [3, 4], but in midline elements (i.e., paraglossal, basihyal, and urohyal) as well (Fig 1). Direct support of the muscular tongue by these bony components is linked to the origin of novel muscles allowing coordination of hyoid and jaw movements during feeding that may also be deployed during panting and vocalization [5, 6]. Rhythmic hyolingual movement coupled with cranial kinesis is critical in avian feeding, especially for a few neognath birds (e.g., Psittaciformes and Anatidae), which are characterized by extensive intra-oral processing of food [3, 7, 8–10]. Extreme morphologies are seen associated with tongue protrusion, involving hyper-elongate paired hyobranchial elements in woodpeckers, hummingbirds, and honeyeaters [2, 11–13].

Muscles are shown in color, while bone/cartilage elements are shown in grey, the airway in birds in white, and fleshy tongue body in pink. Increased mineralization or ossification of hyobranchial elements occurs in ceratobranchial I in Archosauria [1]. Ancestrally within land dwelling vertebrates, the pectoral girdle is the major anchor of the caudal hypobranchial muscles, which function in depressing the hyoid during respiration (insets on left) [49]. Homologous to the episternohyoid complex (ephc) in crocodilians, they are either lost or highly reduced in living birds [37], [50–51]; diminutive tracheal muscles such as M. sternotrachealis and M. tracheolateralis are proposed to be their homologs [51]. These two thin muscles arise from the caudal surface of the craniolateral process on the sternum and syrinx, and insert on the lateral surface of the tracheal wall just cranial to the syrinx and the lateral tracheal wall to the laryngeal cartilages, respectively (left inset, S2 Table; Fig 11). The hypobranchial muscles (mge, mhy, and ephc; dark turquoise) in most non-avian reptiles play an important role in hyoid protraction, retraction, stabilization, and suspension during respiration and feeding [22]. These functions are accomplished by distinct muscles in birds, including M. branchiomandibularis (green), M. stylohyoideus, and M. serpihyoideus (mbm and msh, red color) and associated connective tissues, i.e., hyoid sheath or fasciae [5]. The two antagonistic muscles (mbm and msh) link the hyoid with the lower jaw and facilitate protraction and retraction of the tongue [3]. This major reorganization of hyobranchial muscles is also referred to as hyolingual suspension, which is absent in non-avian reptiles (e.g., *Sphenodon*) [33]. Muscles involved in tongue protrusion are labeled with asterisk. They are also the muscle built in the buccal floor (labeled as pound) in reptiles but lost or reduced in birds. Coordination of the hyoid with the jaw movements during cyclic feeding motions is inferred to be a derived avian feature [3]. Abbreviations: bh, basihyal; ephc, episterno-hyoid complex; mge, M. genioglossus; mhys, M. hyoglossus cranialis and oblique; mbm, M. branchiomandibularis; mca, M. cricoarytenoid; mch, M. cricohyoideus; mhp, M. hypoglossus; msh, M. stylohyoideus and M. serpihyoideus. See Table 1 for specimen information and the tree was adopted from ref. [1]

**Table 1:** Extant specimens and staining protocol applied.

	Taxa and material	Specimen number and sample size (#)	Staining solution (w/v)	Duration	Repository
**Specimen Scanned**	*Alligator mississippiensis* (head)	TNHC specimen (one)	11% I_2_KI	3 days	Texas Natural History Collection
	*Phasianus colchicums* (head)	TMM M-12001	6% I_2_KI	60 days	UT Vertebrate Paleontology Laboratory
	*Dromaius novaehollandiae* (head)	TMM M-12678 TMM M-12679	11% I_2_KI 1% I_2_E	5 days 60 days	UT Vertebrate Paleontology Laboratory
	*Alligator mississippiensis* (tongue)	TMM M-16002	1% I_2_E	10 days	UT Vertebrate Paleontology Laboratory
	*Colinus virginianus* (tongue)	UMNH 23829	0.2% I_2_E	10 days	Natural History Museum of Utah
	*Nothoprocta perdicaria* (head)	NHMU 23838	1–3% I_2_KI	31 days	Natural History Museum of Utah
**Specimen Dissected**	*Alligator mississippiensis*	TMM 12053 and TNHC specimens (three)			UT Vertebrate Paleontology Laboratory; Texas Natural History Collection
	*Phasianus colchicus*	TMM M-12000			UT Vertebrate Paleontology Laboratory
	*Dromaius novaehollandiae*	TMM M-14235, TMM M-14236			UT Vertebrate Paleontology Laboratory
	*Struthio camelus*	TMM M-14237			UT Vertebrate Paleontology Laboratory
	*Aythya americana*	USNM 643740, USNM 643741			National Museum of Natural History, Smithsonian Institution
	*Aythya americana*	TMM M-12682 TMM M-12683			UT Vertebrate Paleontology Laboratory
	*Nothura maculosa*	USNM 631209 USNM 631210			National Museum of Natural History, Smithsonian Institution
	*Rhea americana*	USNM 615363			National Museum of Natural History, Smithsonian Institution
	*Megapodius pritchardii*	USNM 319640			National Museum of Natural History, Smithsonian Institution

In contrast to the diverse tongue morphologies of living birds, their extant sister taxon, Crocodylia, and all extinct basal archosaurs so far studied [14], consistently show a relatively simple hyoid structure [15–17]. Even though a tremendous radiation of Mesozoic Crocodylomorpha (including sphenosuchians) has been reported, their preserved hyoid bones are restricted to a single pair of rod-like ceratobranchials [14]. Megaphagy or hypercarnivory has been proposed as a major cause for the lack of tongue involvement in food acquisition in crocodilians and their extinct relatives [18]. Their broad and fleshy tongue is firmly attached to the buccal floor by muscles and connective tissues. Ossified and cartilaginous elements are small compared to the size of this fleshy tongue. The absence of direct and cranially-extensive support from bony elements make crocodilian tongue incapable of significant independent motion [18]. Relative to outgroup lepidosaurs and other tetrapods the bony structure in crocodilians and surveyed basal archosaurs is uniformly simple and small with a single pair of ceratobranchials and no well-mineralized midline element or fusion [19–22].

While some bony elements of the tongue, or hyoid bones, commonly enter the fossil record [14–18], their shape, and the relationship between muscular and bony components has not been systematically assessed in bird-line archosaurs. Here, we used dissection and diffusible iodine contrast-enhanced computed tomography (diceCT) [23–26] to assess the relationship between bony and muscular features of the tongue in living archosaurs ([18, 27]; Methods). More than 330 fossil specimens, ranging from Triassic stem archosaurs to Jurassic and Cretaceous non-avian dinosaurs and pterosaurs (S1 Table) were examined.

**Fig 2:**
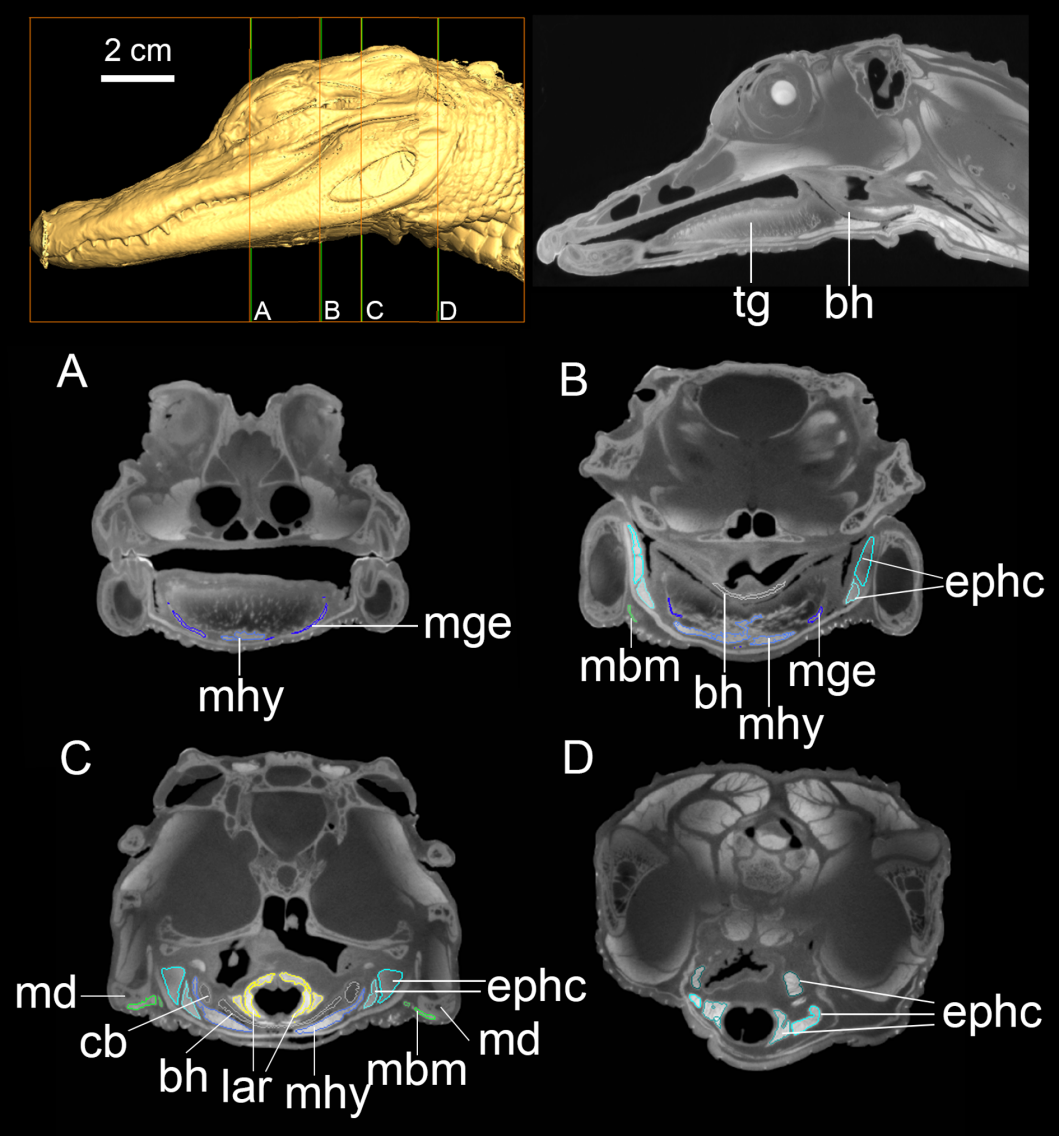
DiceCT imaging of the head of *Alligator mississippiensis*. Individual cross-sections (A-D) from cranial to caudal region with targeted muscular and bony elements colored and labeled. Abbreviations: bh, basihyal; cb, ceratobranchial; ephc, episterno-hyoid muscular complex; lar, larynx; mbm, M. branchiomandibularis; mhy, M. hyoglossus; mge, M. genioglossus; tg, tongue.

**Fig 3:**
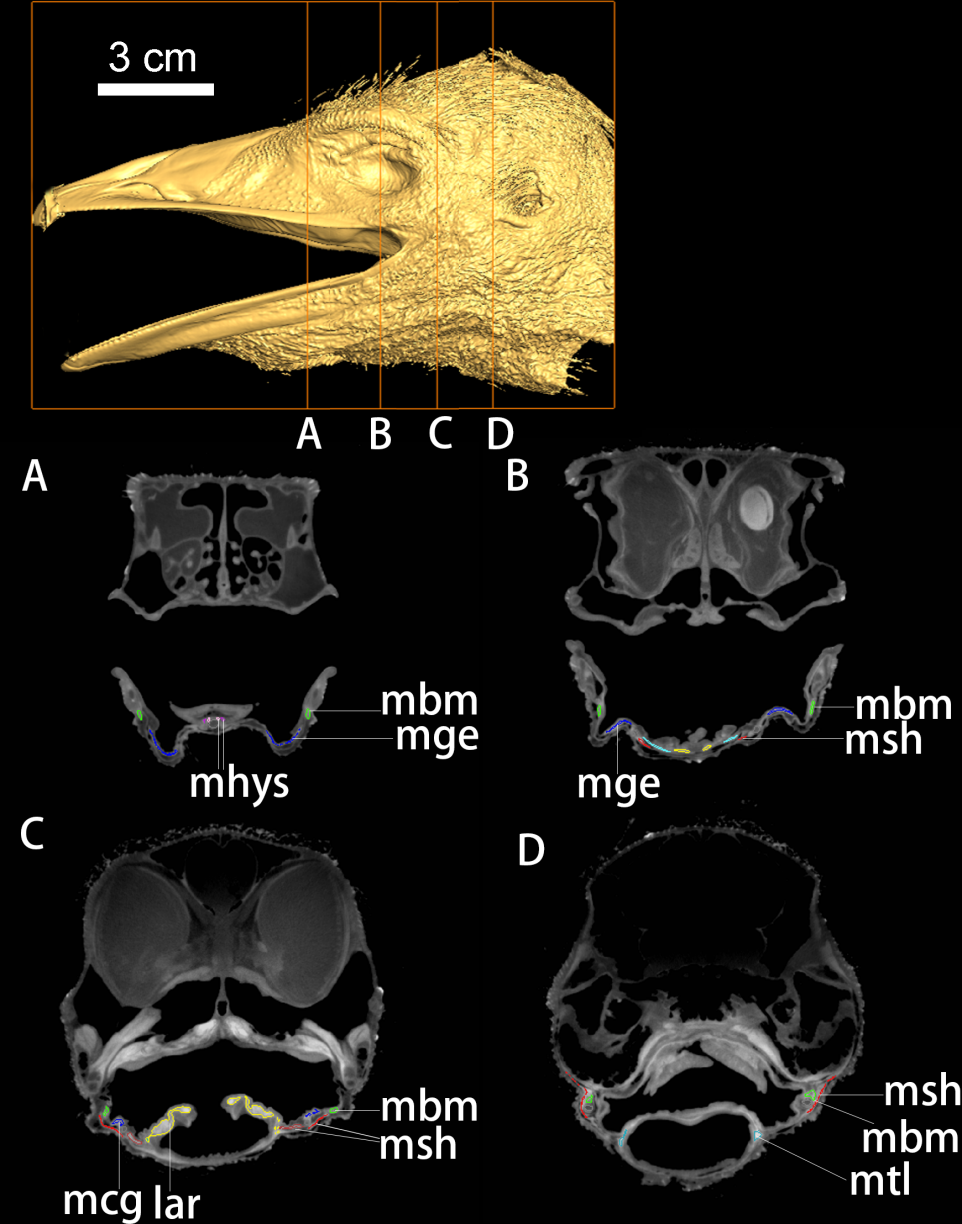
DiceCT imaging of the head of *Dromaius novaehollandiae*. Individual cross-sections (A-D) from cranial to caudal region with targeted muscular and bony elements colored and labeled. Abbreviations: lar, larynx; mbm, M. branchiomandibularis; mcg, M. ceratoglossus; mge, M. genioglossus; mhys, M. hypoglossus; msh, M. stylohyoideus and M. serpihyoideus; mtl, M. tracheolateralis.

**Fig 4:**
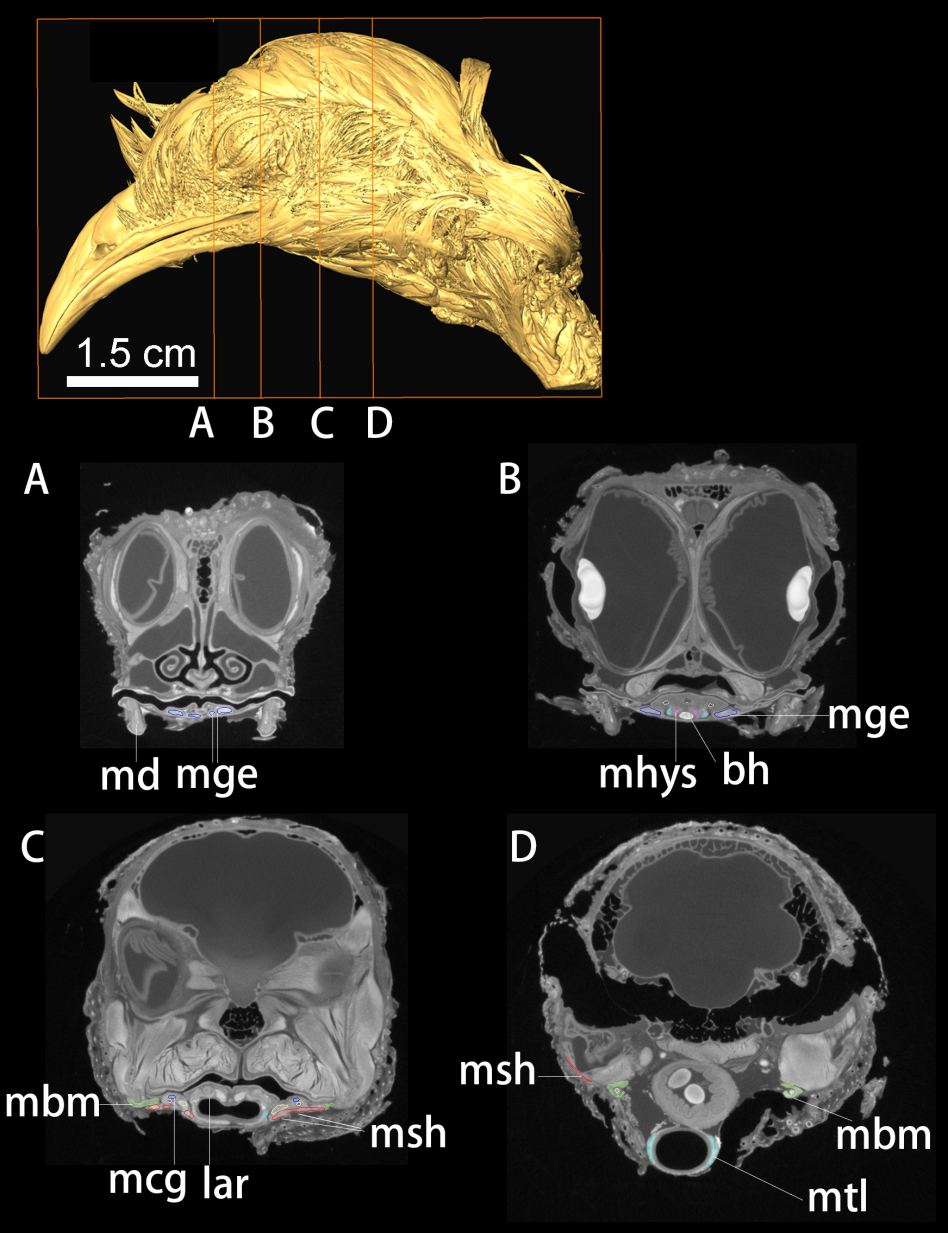
DiceCT imaging of the head of *Nothoprocta perdicaria*. Individual cross-sections (A-D) from cranial to caudal region with targeted muscular and bony elements colored and labeled. Abbreviations: lar, larynx; mbm, M. branchiomandibularis; mcg, M. ceratoglossus; mge, M. genioglossus; mhys, M. hypoglossus; msh, M. stylohyoideus and M. serpihyoideus; mtl, M. tracheolateralis; md, mandible.

**Fig 5:**
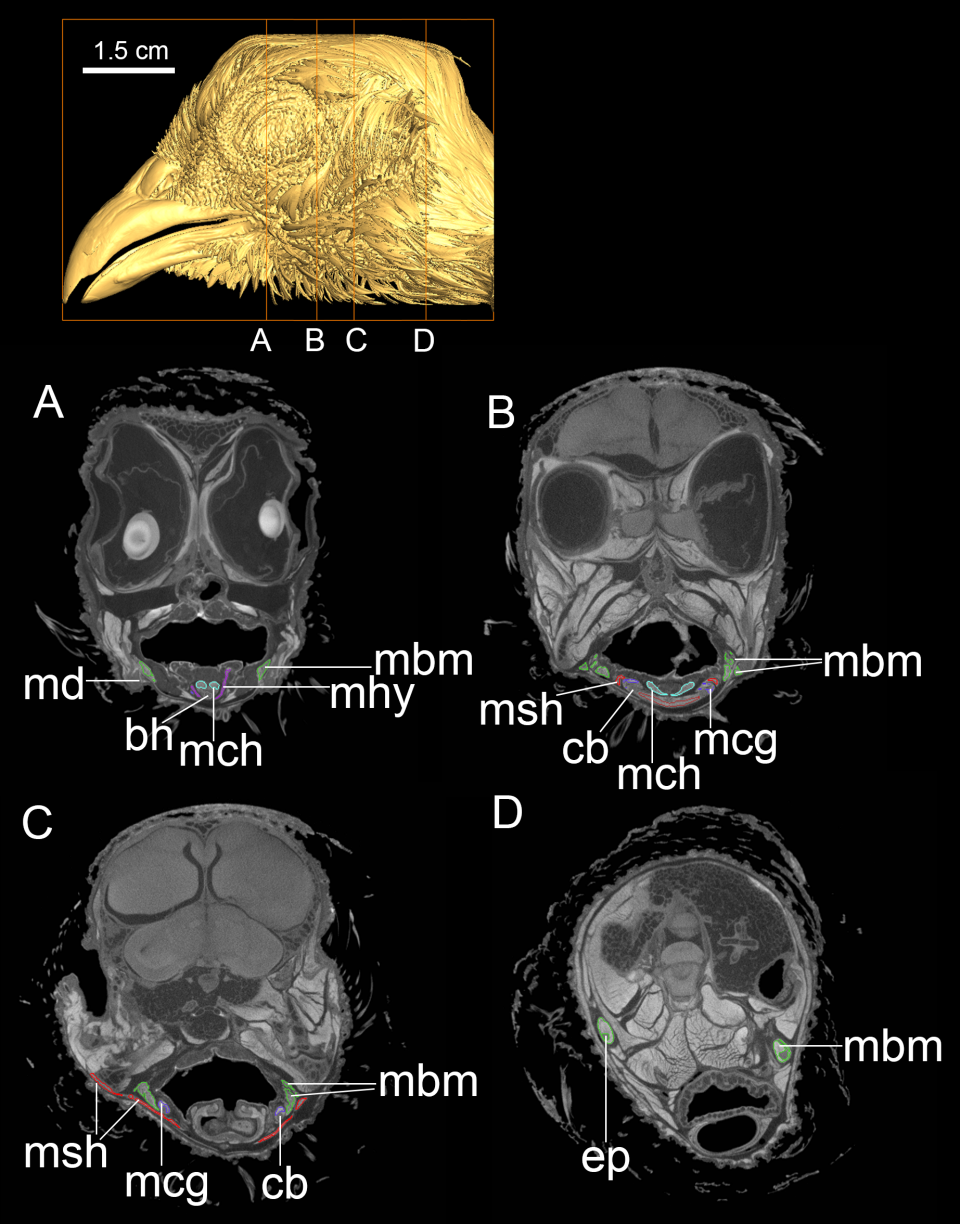
DiceCT imaging of the head of *Phasianus colchicus*. Individual cross-sections (A-D) from cranial to caudal region provided with targeted muscular and bony elements colored and labeled. Abbreviations: bh, basihyal; cb, ceratobranchial; ep, epibranchial; mbm, M. branchiomandibularis; mcg, M. ceratoglossus; mch, M. cricohyoideus; msh, M. stylohyoideus and M. serpihyoideus; md, mandible.

**Fig 6:**
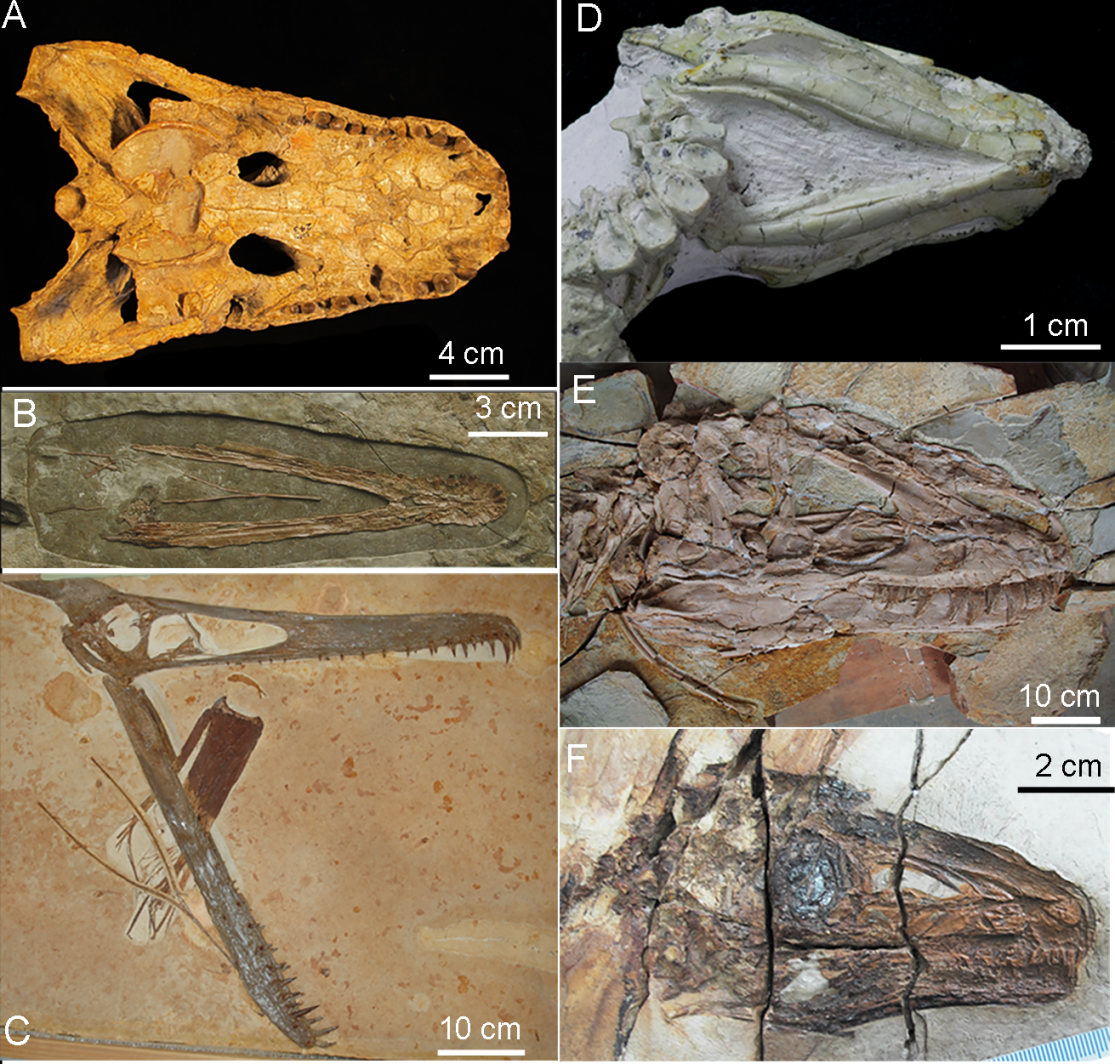
Hyoid remains in extinct archosaurs. (A)*Alligator prenasalis*, pterosaurs (B)*Liaoxipterus brachycephalus* and (C)*Ludodactylus sibbicki*, (D)basal ornithischian *Jeholosaurus shangyuanensis*, (E)tyrannosaur *Yutyrannus huali* (F) *Sinosauropteryx prima*.

**Fig 7:**
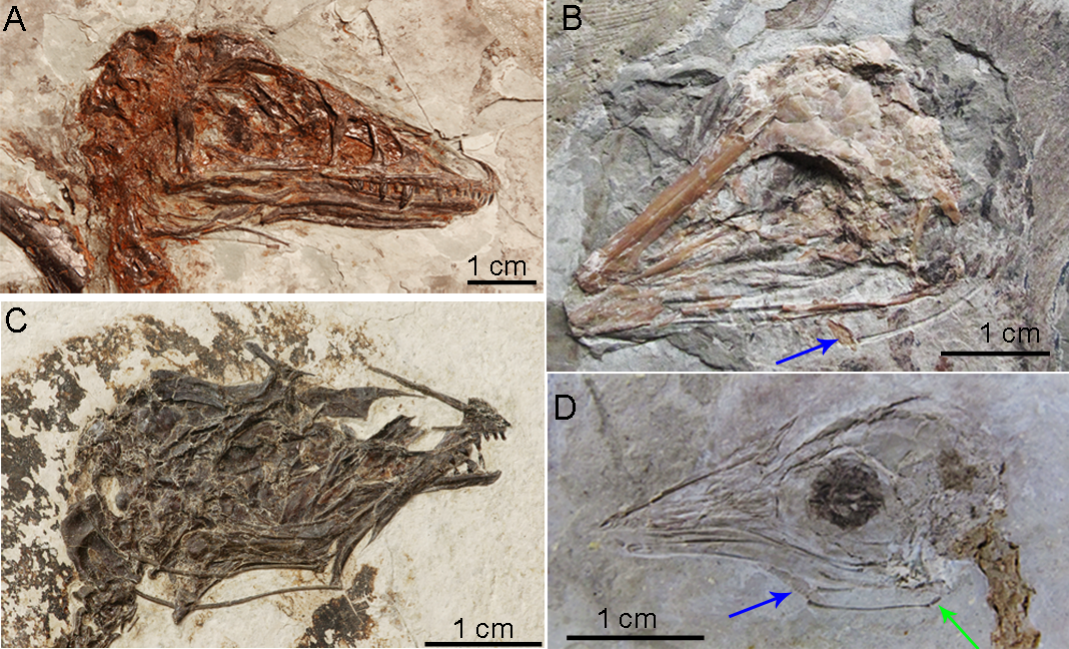
Hyoid remains in paravians. (A) *Microraptor zhaoianus*, (B)*Confuciusornis sp*., (C)*Enantiornithine* sp., (D)*Hongshanornis longicresta*. The blue arrow indicates the ossified basihyal in *Confuciusornis* and *Hongshanornis*; it was also observed in one specimen of *Microraptor*. The green arrow indicates the phylogenetically earliest epibranchial [15]. See S1 Table for specimen numbers and supplemental references for published specimens.

We detailed hyolingual muscles from exemplars of birds from Neognathae and Palaeognathae, and used these with comparison of outgroup taxa to inform estimation [28] of ancestral hyolingual features of Aves (Methods; supporting material). Identification of key soft-tissue correlates required comparison of avian hyoid features with those of outgroup reptiles. The anatomy and function of the tongue in extant crocodilians and inferred implications for basal archosaurs has already been recently treated [18]. Outgroup comparison was used to determine the ancestral hyoid condition for archosaurs and assess derived hyolingual features of birds [18, 28]. This work underpins and constrains the inference of shifts in tongue function in Archosauria and provide key insight into the possible co-evolution of tongue morphology and feeding ecology (Figs 1–7; Methods) [18, 23, 29, 30].

## Methods

### Specimen preparation and fossil sampling

Extant specimens were prepared and dissected at the Texas Natural History Collections (TNHC) at the University of Texas at Austin. USNM-catalogued specimens were dissected and photographed at the Museum Support Center at the Smithsonian Institution, National Museum of Natural History (NMNH), Washington DC. All specimens were scanned at the University of Texas High-Resolution X-ray Computed Tomography Facility (UTCT). The protocols of iodine staining for soft-tissue contrast imaging were adapted from methods successfully developed for alligator and birds [18, 23, 29, 31, 32].

All specimens were fixed in 10% neutral buffered formalin solution (10% NBF) for approximately a week to two months based on the specimen size. Individual specimens were stained with either I_2_KI (iodine-potassium iodine-10% NBF solution) or I_2_E (iodine-ethanol solution). The concentration of the I_2_KI solution was calculated by dividing the total weight of solutes (e.g., iodine and potassium iodine) by the volume of the solution. Alternatively, 1% I_2_E (1g I_2_ dissolved in 100 ml absolute ethanol [200 proof]) was used in staining the tongues of American alligator and Northern bobwhite (*Alligator mississippians* and *Colinus virginianus*) The concentration of staining solution, duration of staining and scanning parameters are detailed in the Tables 1 and 2. All smaller samples, including Ring-necked Pheasant (*Phasianus colchicus*), Chilean tinamou (*Nothoprocta perdicaria*), and Northern bobwhite (*Colinus virginianus*) were scanned using a microXCT 400 scanner (built by Zeiss, formerly Xradia, Inc.). The larger *Dromaius novaehollandiae* (Common Emu) were scanned using a BIR scanner (225 kV Feinfocus X-ray source and an Image Intensifier detector) in UTCT.

**Table 2:** Scanning parameters for extant specimens.

Species	Specimen number	Images	Voltage (kV)	Current (mA)	Slice thickness (mm)	Inter-slice spacing (mm)	Field of reconstruction (mm)	No. of slices
***Alligator mississippiensis* (head)**	TNHC specimen	1024x1024 16-bit	150	0.4	0.08638	0.08638	82	1538
***Dromaius novaehollandiae* (head)**	TMM M-12678	1024x1024 16-bit	150	0.4	0.1115	0.1115	106	1362
***Colinus virginianus* (tongue)**	UMNH 23829	1024x1024 16-bit	70	0.14	0.03581mm (voxel size)			2360
***Alligator mississippiensis* (tongue)**	TMM M-16002	1024x1024 16-bit	150	0.13	0.126mm (voxel size)			1732
***Phasianus colchicus* (head)**	TMM M-12001	1024x1024 16-bit	120	0.08	0.04789mm (voxel size)			1468
***Nothoprocta perdicaria* (head)**	NHMU 23838	1024x1024 16-bit	120	0.08	0.04239mm (voxel size)			1518

**Fig 8:**
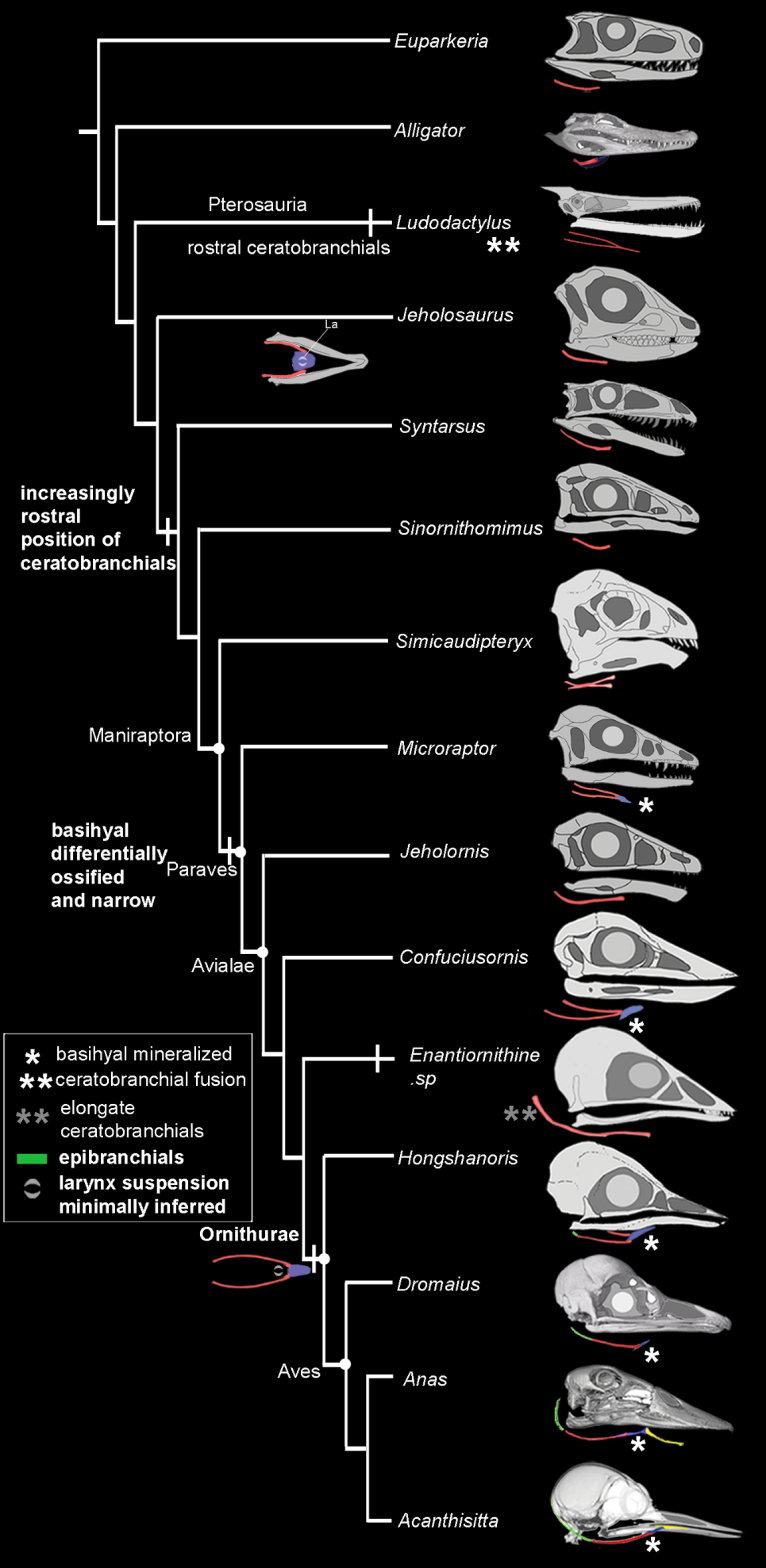
The evolution of the tongue in Archosauria.

Fossil data were systematically assessed from the Shandong Tianyu Museum of Nature (n ~250) and Institute of Vertebrate Palaeontology and Palaeoanthropology (n ~50), and Beijing Museum of Natural History (n ~30). The best-preserved representative specimens are cited in the S1 Table. Reconstructions of extinct archosaur skulls and hyoids (Fig 8) were based on examination of fossil material in museum collections (Institute of Vertebrate Paleontology and Paleoanthropology, IVPP and Shandong Tianyu Museum of Nature, STM), Beijing Natural History Museum, as well as published resources (S1 Table). For all extinct archosaurs, the resting position of hyoid relative to the skull (i.e., the eye orbit) was assessed as similar to that preserved unless there was clear evidence of displacement post mortem.

Pterosaurs show convergent evolution of traits linked to tongue protrusion and mobility in birds (narrow midline element [achieved through fusion] and elongate, paired and rostrally positioned ceratobranchials). Within Dinosauria, hyoid elements are progressively more rostrally-placed in crownward bird-line species, a condition particularly evident in extant birds (compare orbital position). The ceratobranchial-basihyal contact is approximately even with the nasofrontal hinge (or zone of contact between the premaxillae and frontals) in birds, which is rostral to its position present in successive outgroups. A second ceratobranchial is absent in all archosaurs with the exception of one proposed example in Ankylosauria [10, 41]. However, unlike outgroup taxa, the position of ceratobranchial I in archosaurs is rostrally displaced relative to the center of the orbit. Skeletal hyoid elements are highlighted in different colors: red, ceratobranchial; blue, basihyal; yellow, paraglossal; green, epibranchials. References of reconstructions used are provided in S1 Table and the tree was adopted from ref. [52].

Institution abbreviations: BMMS, Bürgermeister Müller Museum Solnhofen; BMNH, Beijing Museum of Natural History; DNHM, Dalian Natural History Museum; ELDM, Erlianhaote Dinosaur Museum, Inner Mongolia; IVPP, Institute of Vertebrate Palaeontology and Palaeoanthropology; MNA, Museum of Northern Arizona; MACN, Meseo Argentino de Ciencias Naturales; NIGP, Nanjing Institute of Geology and Palaeontology; PKUP, Peking University Paleontological Collections; SAM, South African Museum; XHPM, Xinghai Museum of Prehistoric Life of Dalian; SDSM, South Dakota School of Mines and Technology; SMNK, Staatliches Museum Für Naturkunde Karlsruhe; STM, Shandong Tianyu Museum of Nature; TNHC, Texas Natural History Collection; UMNH, Natural History Museum of Utah; USNM, the National Museum of Natural History, Smithsonian Institution; UT-TMM, University of Texas at Austin, Vertebrate Paleontology Laboratory; ZDM, Zigong Dinosaur Museum, Zigong, China; ZLJ, Lufeng World Dinosaur Valley Park (see also S1 Table).

### Image processing

All 16-bit images were imported into Avizo 6.1 for the segmentation of bony hyoids and major hyoid muscles in *Dromaius*, *Nothoprocta*, *Colinus*, *Phasianus*, and *Alligator* (Figs 1–4). The segmentation of a particular muscle or a bony element was made by adjusting the grayscale of image contrast until distinctions were sufficient for automatic selection by the software based on the contrast (Figs 2–5). When targeted muscular or cartilaginous tissues cannot be selected automatically due to the diminished boundary, the paint-tool was used for manual selection in Avizo 6.1. In general, muscular tissues were much higher in grayscale value than other tissues measured (fasciae and other close tissues bounded the muscle; Figs 2–5), with cartilage and other connective tissues (e.g., fasciae) being the lowest. Interpretation of specific muscular anatomy was further validated through dissection (Table 1). SurfaceGen (in Avizo 6.1) was used to render the 3-D structures of bony hyoid and muscular tissues.

Skull images were adopted from ‘digimorph.org’ (Fig 1: *Glyptemys muhlenbergii*, *Sphenodon punctatus*, and *Alligator*) or were based on available CT data (*Acanthisitta chloris*, *Nothoprocta perdicaria*). The mandibles of *Dromaius* and *Phasianus* are photos of NMNH specimens. Images of the skull, mandible, and digital reconstructions of hyoid and muscular tissues were composed in Photoshop CS5. Their relative positions were based on careful evaluation of CT data. Schematic diagrams of hyoid muscles in *Sphenodon* and *Glyptemys* were reconstructed based on ref [19, 33].

### Ancestral state estimation for the hyoid features investigated

Ancestral state estimation for muscular and bony features was undertaken in using a parsimony estimator [34]. Recovered character optimizations were described in supporting material. Results are also shown in Figs 1 and 8 and S1 and S2 Figs). We found unambiguous character changes based on ancestral character state reconstruction using parsimony, for Palaeognathae (character 13) and Neognathae (characters 10 and 30), indicating divergence in tongue morphology related hyoid traits in extant birds. Changes reconstructed within the clade (Fig 8; supporting information) are minimum estimates of shifts.

## Results

### Similarity in hyobranchial morphology across most of non-avian archosaurs

**Fig 9:**
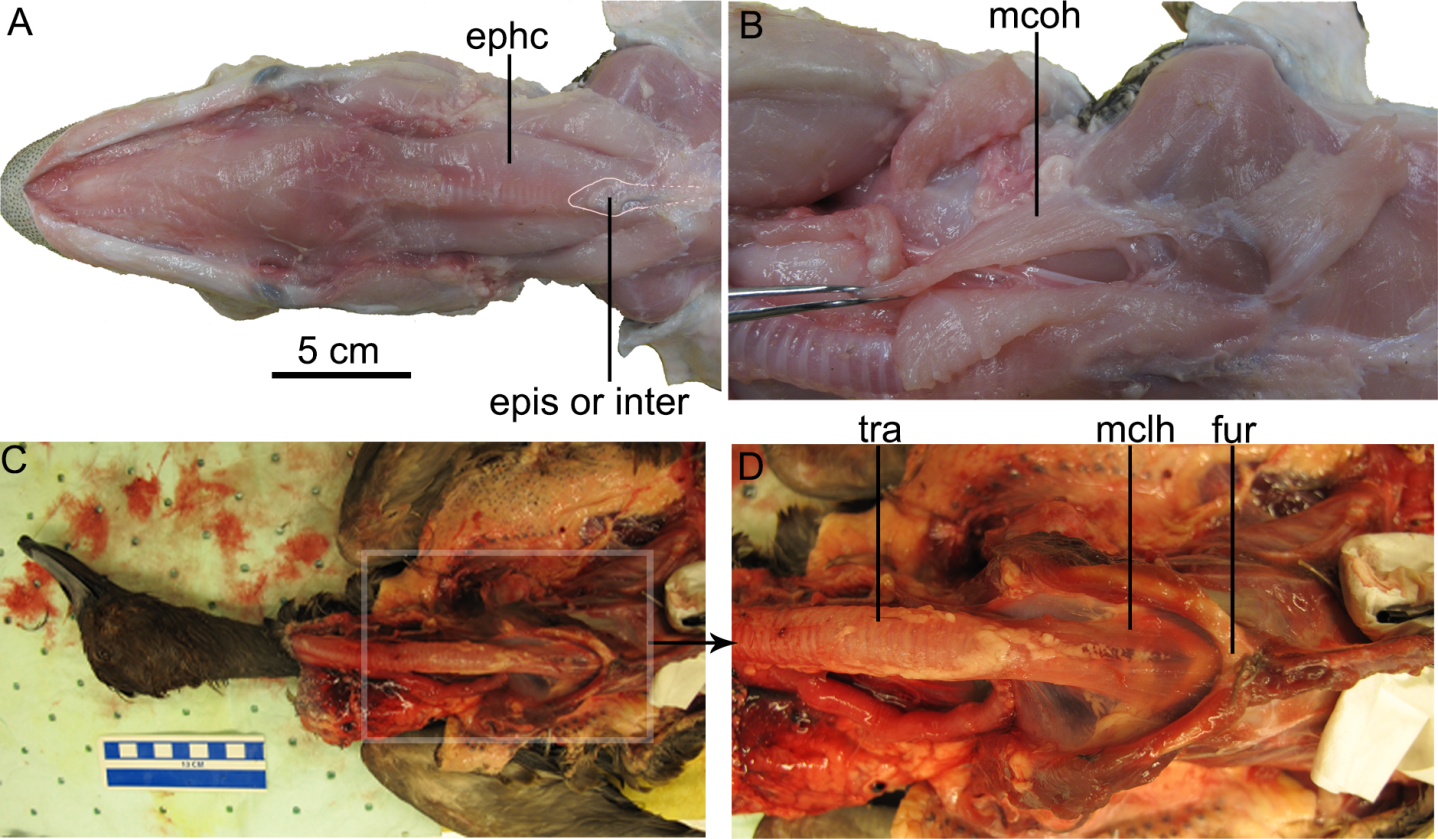
Episterno-hyoid and M. coracohyoideus muscle in *Alligator mississippiensis* and proposed homologous muscles in *Aythya americana*. (A)(B)*Alligator mississippiensis* (TMM M-12053), (C)(D)the homologous M. sternotrachealis in *Aythya americana* (TMM M-12682). Abbreviations: ephc, episterno-hyoid complex; epis or inter, episternum or the interclavicle; fur-furcula; mclh, M. cleidohyoideus (in *Aythya*); mcoh, M. coracohyoideus (in *Alligator*); tra, trachea.

**Fig 10:**
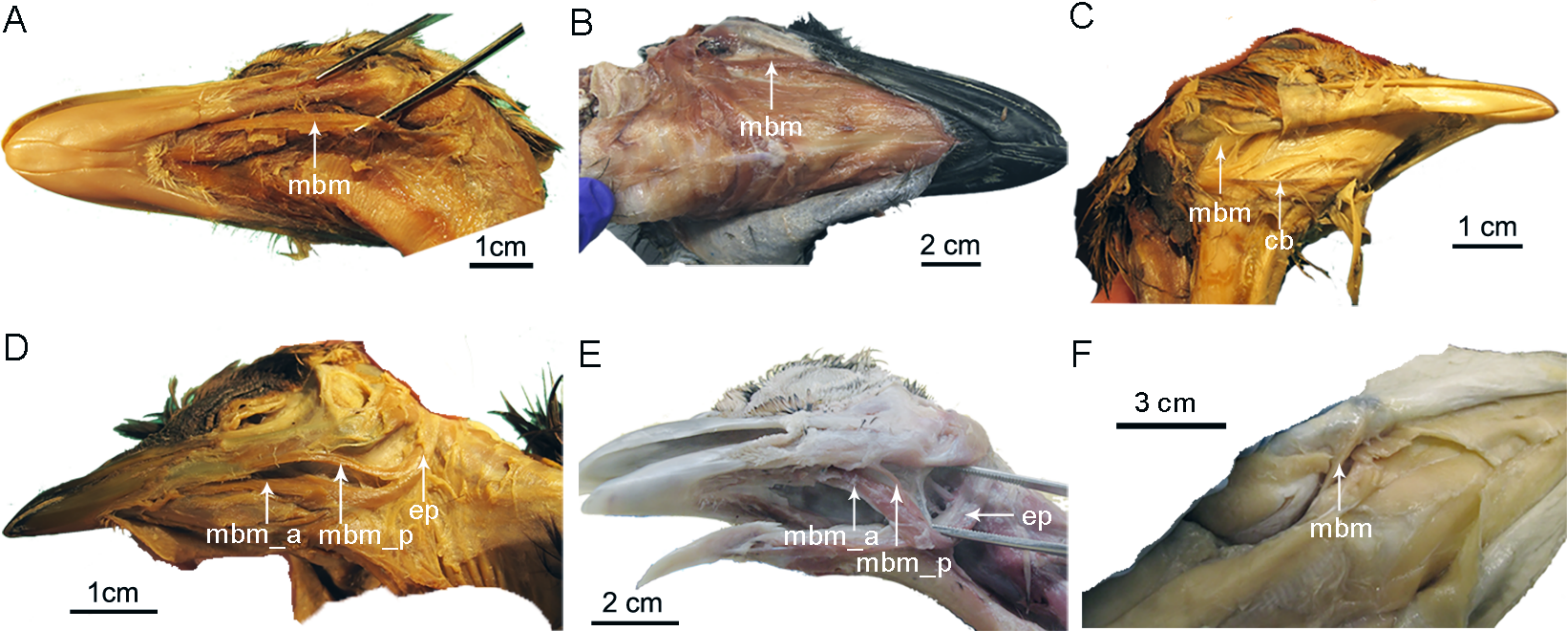
The M. branchiomandubularis (arrows) in birds and *Alligator*. (A)*Rhea americana* (USNM 615363), (B)*Dromaius novaehollandiae* (TMM M-14235), (C) *Nothura maculosa* (USNM 631209), (D)*Megapodius pritchardii* (USNM 319640), (E) *Phasianus colchicus* (TMM M-12000), (F)*Alligator mississippiensis* (TNHC specimen). The two portions of the muscle are indicated in Galliformes: ‘M. branchiomandubularis cranialis’ (mbm_a) and ‘caudalis’ (mbm_p).

**Fig 11:**
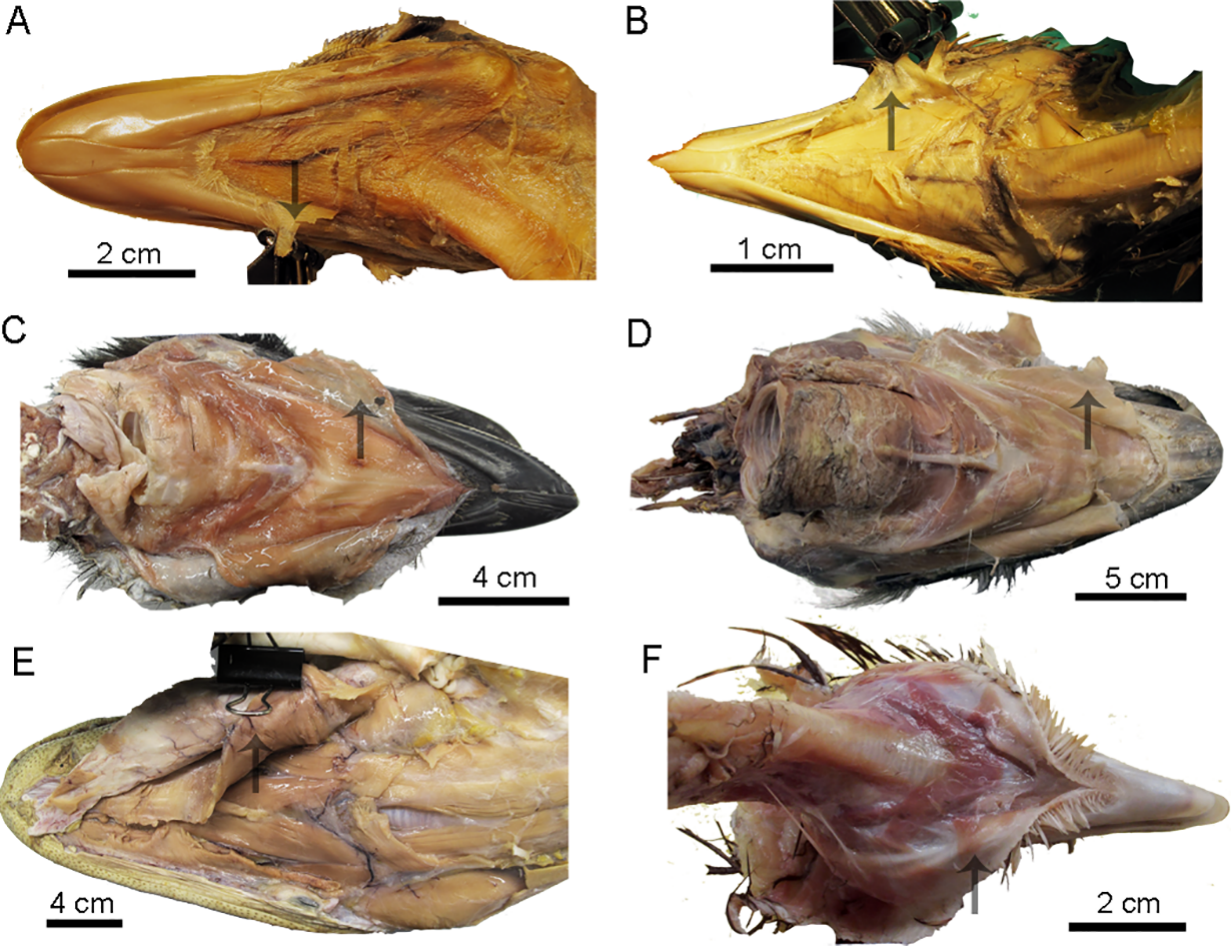
M. intermandibularis cranialis in birds and *Alligator*. (A)*Rhea americana* (USNM 615363), (B)*Nothura maculosa* (USNM 631209), (C)*Dromaius novaehollandiae* (TMM M-14235), (D)*Struthio camelus* (TMM M-14237), (E)*Alligator mississippiensis* (TNHC specimen) and (F)*Phasianus colchicus* (TMM M-12000). The muscle was indicated by black arrow.

**Fig 12:**
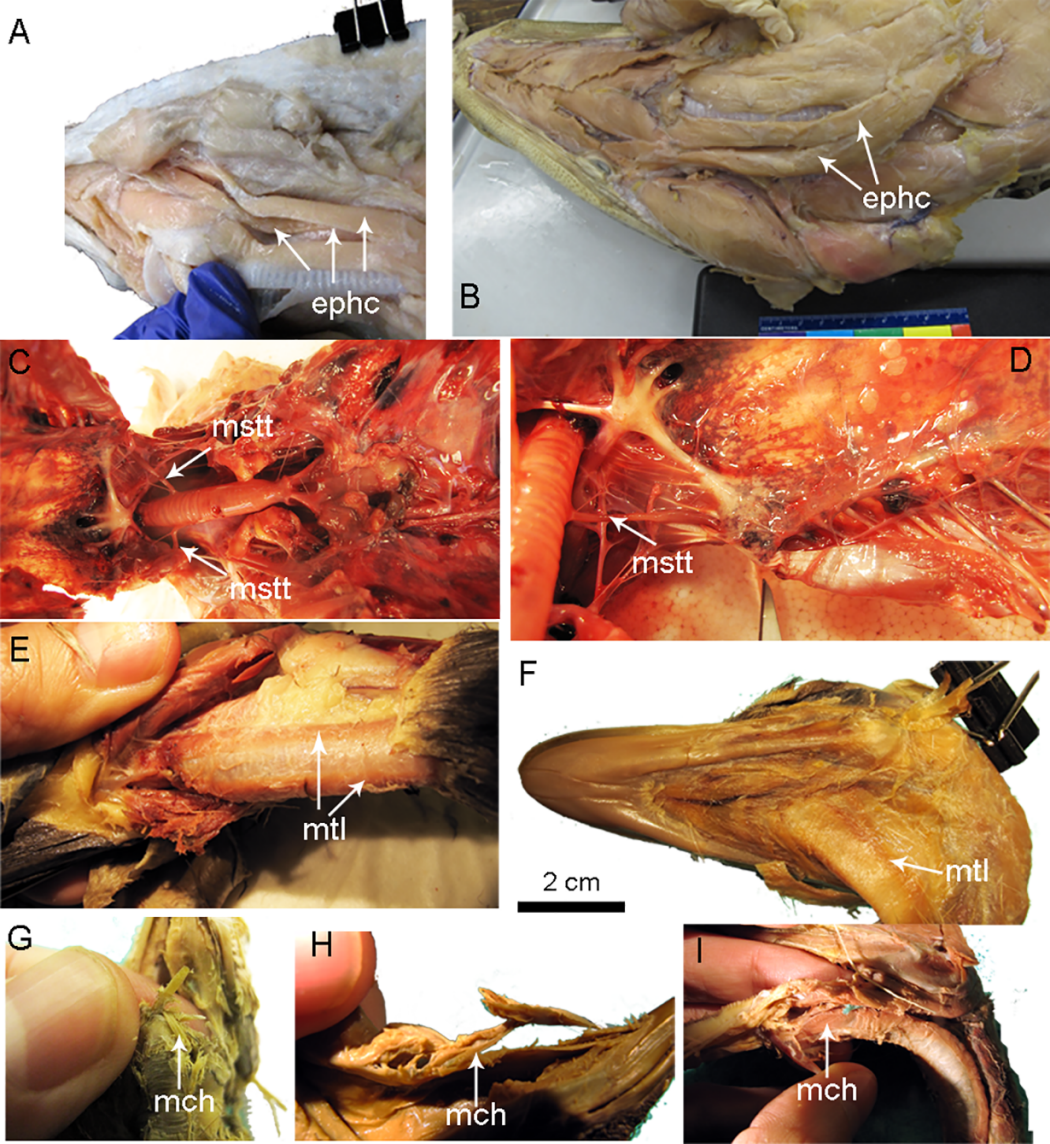
Episterno-hyoid and M. coracohyoideus muscle in *Alligator* and proposed homologous muscles in birds. (A)(B)*Alligator mississippiensis* (TMM M-12053), (C)(D)the homologous M. sternotrachealis in *Aythya americana* (TMM M-12682). (E)(F) show the M. tracheolateralis in *Aythya americana* (USNM 643741) and *Rhea americana* (USNM 615363) respectively; (G)(H)(I) show M. cricohyoideus in *Nothura machulosa* (USNM 631210), *Megapodius pritchardii* (USNM 319640) and *Aythya americana* (USNM 643741) respectively Abbreviations: ephc, episterno-hyoid complex; mch, M. cricohyoideus; mclh, M. cleidohyoideus; mtl, M. tracheolateralis, and mstt, M. sternotrachealis.

The hypobranchial muscles are major connections of the hyoid caudally to the pectoral girdle and cranially to the mandible (Fig 1). The loss of episternum occurs in Ornithodira, reduced the tight caudal link between the hyoid and pectoral girdle (Figs 1 and 9). These muscles attach to the ceratobranchial elements (Fig 1). Cranial portions of the hypobranchial muscles compose part of a muscular pad in the fleshy non-avian reptile tongue (e.g., M. geniohyoideus and M. hyoglossus), but are also largely reduced in birds (Figs 1 and 10–12). They are also the major tongue protractor in reptiles; by contrast, birds have a pair of distinct hyobranchial muscle (M. branchiomandibularis) that functions in tongue protraction (Figs 1 and 10).

Reduction of the hypobranchial muscles and presence of a single set of thin rod-like ceratobranchials (Fig 1), has been previously reported to have arisen early, beginning in stem lineage archosaurs [14, 18] associated first with respiratory innovations and a reduced role for depression of the hyoid during respiration via buccal pumping [18, 33, 35]. Basal archosaur fossils uniformly show a single set of these structures and no evidence of ossification of a midline element. We find pterosaurs similarly show lightly-built single pairs of ceratobranchial elements (Figs 6 and 7). This trend continues within Dinosauria where a single set of paired elements are thin in all but some ornithischians (Figs 1 and 6; S3 Fig). In Theropoda, from *Carnotaurus* [17] through oviraptorosaurs, the ceratobranchials are thin, short, and caudally positioned (Fig 8) while in a resting position.

New retractors of avian hyolingual apparatus include the M. serpihyoideus and M. stylohyoideus (Figs 1–5; [36]), both these muscles have not been observed in *Alligator* or other closely-related outgroup (e.g., *Sphenodon*). The two muscles extend from the distal caudal end of the mandible on ventral side (Fig 1), and extend cranially attaching on the fascia that linked to the ventral and dorsal aspect of the urohyal and basihyal respectively (Figs 1 and 3–5). The weakened ancestral function for the hypobranchial muscles in hyoid retraction might drive the acquisition of novel avian hyoid retractor (Figs 1 and 5). However, we could not identify proxies to inform the timing of these shifts in extinct taxa.

### Midline elements, evolution of the paraglossal, and intrinsic musculature

At least two well-ossified midline elements, the basihyal and paraglossal, are present in all neognath birds but are incompletely or variedly ossified in paleognaths (Figs 3–5). By contrast only a single cartilaginous midline element, a basihyal, is present in extant crocodilians and turtles [18]. The broad and cartilaginous basihyal is comparatively caudally-positioned in crocodilians; while more cranially-positioned (relative to the eye orbital position), ossified midline elements are associated with reduced fleshy tongue size and increased mobility in Aves (Fig 1).The avian tongue is characterized by origin of associated intrinsic tongue muscles, such as the M. hypoglossus (Fig 1: mhps) [36, 37], associated with the position, shape, number and ossification of the unique midline lingual elements, i.e. basihyal and the paraglossal (Figs 3–5).

We observed only a single element, consistent with an ossified basihyal within Paraves (i.e., a single specimen of *Microraptor*; multiple specimens of *Confuciusornis* and *Hongshanornis longicresta*; Figs 6 and 7; S4 and S5 Figs) [15, 16]. Midline ossification was also previously hypothesized for the more basally-divergent theropod, *Carnotaurus sastrei* [17]. However, as described it is not similar to paravian basihyals; it is blunt in *Carnotaurus* [17], rather than tapered, and may be derived in that taxon. A basihyal is unknown in all other non-paravian theropods and most basal Paraves. Although no midline element ossifications are found in pterosaurs, some show a rostral fusion [38] of the ceratobranchials unique in Archosauria (Figs 6B, 6C and 7). In derived ornithischian dinosaurs (e.g., ankylosaurids and hadrosauroids, [39–41]), midline elements are sometimes ossified [14, 41], but are mediolaterally broad and sometimes associated with a second set of paired elements more similar to the condition in turtles but not known in any extant archosaurs [18] (Fig 1).

Given the condition in paleognathous crown taxa and the fossils considered and the absence of fossil evidence of a paraglossal within all non-crown avian theropods, the muscular linkage of this element to the basihyal seen in neognaths, is estimated to occur within the avian crown clade (Fig 8) paleognathous birds. We found no evidence of a well-ossified paraglossal in any non-avian dinosaurs (contra ref. [14] on a derived ornithischian). However, we cannot exclude a cartilaginous presence of this novel element that might occur earlier in non-avian dinosaurs and secondarily reduced in these crown avian taxa.

### Hyobranchial elongation, midline morphology and contact, and tongue mobility

In extant crocodilians, the large fleshy tongue occupies most of the rostral buccal cavity and is firmly attached to the buccal floor (Figs 1 and 2). Therefore, the tongue mobility and protrusion (cranio-caudal motion) is limited and only weakly visible during contraction of the cranial hypobranchial muscles [42, 43]. Although the tongue is similarly largely attached to the buccal floor in tortoises as well, more lingual mobility has been observed in the feeding process in these taxa [1]. A broad, better-mineralized basihyal, with a small lingual process attached to it ventrally, distinguishes these tortoises from archosaurs [1]. In contrast, a mobile tongue capable of significant rostro-caudal movement or protrusion is present in all members of Aves [3, 44].

The elongate, cranio-caudally extended branchiomandibular muscles in birds extend cranially along the length of the hyobranchial elements, i.e., ceratobranchials and epibranchials, to the mandible (Figs 1, 3 and 5) and play a novel role in protrusion during avian feeding (Fig 1; [4]). Distinct mechanisms and tongue muscles are used in the protrusion of a completely fleshy rather than a bony tongue in lepidosaurs (e.g., iguanians) and frogs [1, 2]. Short, caudally positioned ceratobranchials with mediolaterally extensive branchiomandibular muscles are present in *Alligator*, where they facilitate largely dorsoventral movement [20].

A gradual trend in Dinosauria is seen towards a more rostral location of the ceratobranchials and the midline element they cranially contact relative to the cranial edge of the orbit (Fig 8). The earliest known ossified epibranchials are seen in the ornithurine bird *Hongshanornis* [11] (Fig 7, S4 and S5 Figs). However, we observed significant elongation of ceratobranchial elements, in pterosaurs (e.g., *Liaoxipterus brachycephalus* and *Ludodactylus sibbicki*) and some enantiornithine birds (e.g., *Sulcavis geeorum* and an unnamed species, IVPP V13266). This trait varies independently of the presence of an ossified basihyal (in Dinosauria) or ceratobranchial fusion (in Pterosauria; Fig 6). It appears to be convergent on elongation of the hyobranchial apparatus achieved via a separate epibranchial in ornithurine birds including Aves (Figs 6 and 7).

### Larynx position

**Fig 13:**
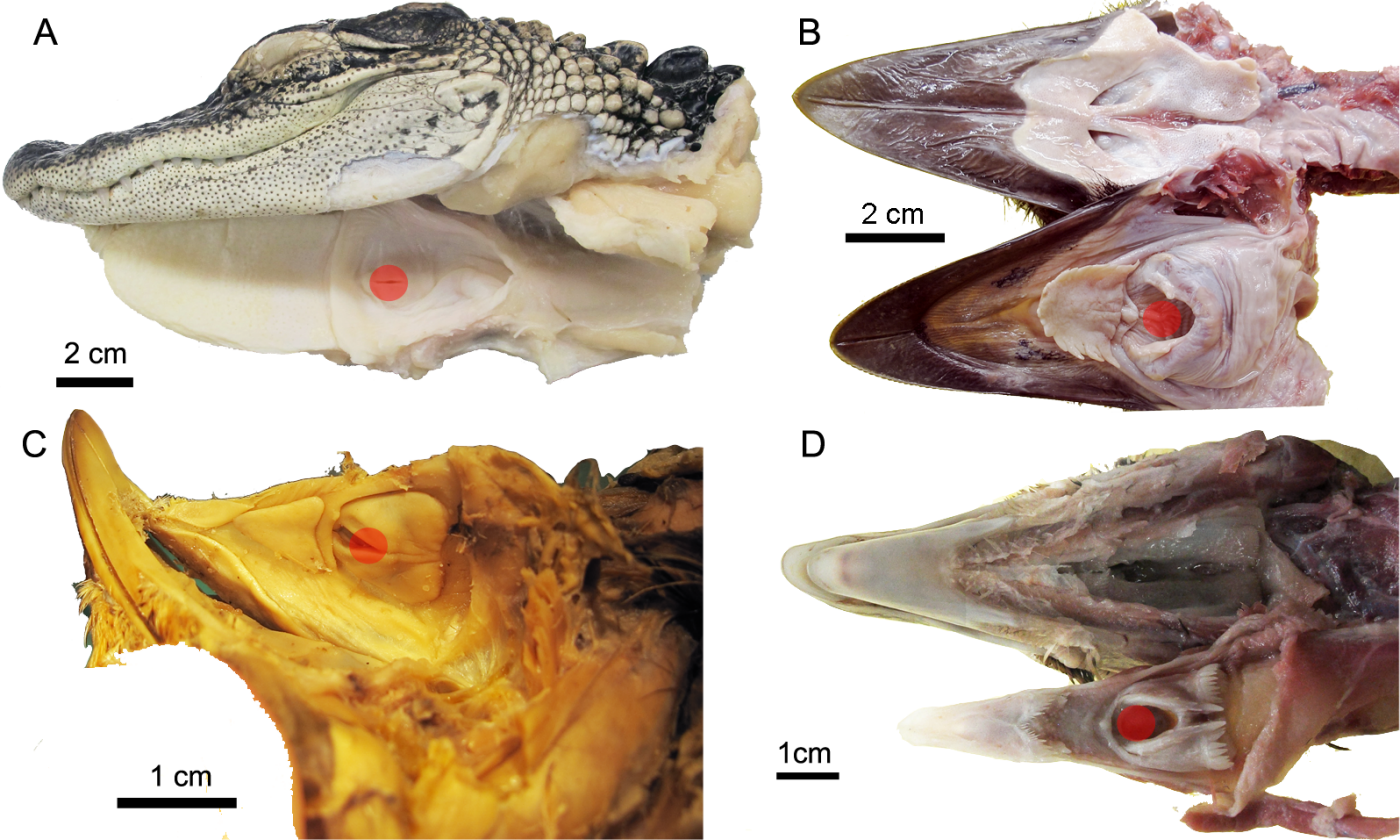
The position of the laryngeal opening in birds and *Alligator*. **Laryngeal opening is indicated by the red circle.** (A)*Alligator mississippiensis* (TNHC specimen), (B)*Dromaius novaehollandiae* (TMM M-14236); (C)*Nothura maculosa* (USNM 631210), (D)*Phasianus colchicums* (TMM M-12001).

We noted a striking further transition with Archosauria in the position of the larynx relative to the hyoid and tongue (Fig 13). In all extant non-avian reptiles, the opening of the airway or larynx lies directly on, or cranial (e.g., snakes) to, the basihyal regardless of whether that element is well ossified or just cartilaginous (Fig 1: lar). By contrast, the highly-mobile avian larynx is always positioned on the urohyal, suspended caudal to an ossified basihyal by an avian hyoid muscle, M. cricohyoideus (Figs 1 and 12: mch). The muscle extends from dorsal surface of basihyal and attaches to ventral surface of the cricoid bone (Fig 1). This muscle is well-developed in neognath birds, where it is described as coordinating laryngeal movement with the hyoid during swallowing [7]. It is weakly developed in paleognaths [7] in which coordination during swallowing is arguably no less important but remarkedly different [3]. It is possible that basihyal and urohyal ossification and cranially-located midline contact between the ceratobranchials and basihyal may indicate this shift has occurred. However, we could not confidently identify a single proxy to inform the timing of this shift in extinct archosaurs.

## Discussion

The bony tongues of living birds share midline elements and paired epibranchials that facilitate feeding ecologies from nectivory to filter feeding and piscivory [2, 8, 9, 14, 44]. Additional complex keratinous structures on the surface of the tongue that as diverse as groves, spines, and complex ridges (e.g., quails, ducks, and penguins; [11, 45]), are not known to have a fossil record [46]. The fossil record of bony hyoids has indicated a simplification of the tongue preceding the origin of the common ancestor of crocodilians and birds related to the loss of a primary respiratory function of the hyoid [18]. While non-avian archosaurs diversified in body size and dentition, most do not show elaboration of the hyoid apparatus, at least based on fossil evidence (Figs 6 and 7). Within archosaurs, only derived herbivorous ornithischian dinosaurs and taxa that evolved flight, tongue structure appears to be uniquely elaborated (Figs 6–8).

### Elaboration of dinosaurian hyoid elements

The elongation of hyobranchial elements (ceratobranchials and/or epibranchials), which are associated with elongation of branchiomandibular muscles, rostrocaudal movement (protrusion) and tongue mobility [1, 8, 9, 18] are exclusively seen in volant taxa where it is estimated to have arisen independently three times within archosaurs. Two different mechanisms are seen, elongation of the ceratobranchial pair in pterosaurs and several enantiornithine birds as well as origin of a separate set of ossified epibranchials articulated to the caudal end of the ceratobranchials uniquely in ornithurines including Aves (Figs 6 and 7). The origin of the ornithurine epibranchials arises coincident with other inferred shifts in rostral shape [47] should be investigated as potentially related to a proposed reorganization of facial development [47]. Functionally, rostrocaudal movements in associated with food manipulation [1, 8, 9, 18] and may single selective pressure in volant lineages for repositioning prey items prior to swallowing in the absence of more dexterous forelimbs that are now constrained by competing selective pressure related to a function locator apparatus.

Elongation of hyobranchial elements co-occurs with increased ossification of a midline element (i.e., in paravians) and ceratobranchial fusion on the midline (i.e. pterosaurs). A trend toward increasing ossification of a single midline element, the basihyal is seen starting within basal Paraves may imply a response to a shift in tongue function acquired earlier. The condition in both clades is marked contrast to the widely-spaced elements in crocodilians and basal ornithischians for example (Figs 6 and 7). It is also well mineralized in some primarily quadrupedal, herbivorous ornithischians dinosaurs (e.g., ankylosaurids and hadrosauroids [39, 41]). Within testudines, increase ossification of the midline element is seen in terrestrial taxa with an increase role for intraoral manipulation of food by the tongue [1]. This aspect of hyoid elaboration in archosaurs we propose may again be linked to tongue mobility in acquisition and intraoral processing in response to diet shifts and diminished utility of the forelimbs in food manipulation.

Within Dinosauria, the origin of new avian intrinsic tongue musculature may be present minimally within Paraves signaled by ossification of the basihyal and a more cranially positioned contact of this element with the ceratobranchial (Fig 8; S4 and S5 Figs). However, we were able to identify strongly supported proxies for this shift and its timing should be considered tentative (Fig 8). There is no evidence for a second midline ossified element homologous with the avian paraglossal (which serves as an additional attachment site for intrinsic muscles) in any non-crown clade avialans, or Theropoda. This element is only weakly mineralized in some paleognaths. The complex hyoid morphology of some ankylosaurs (Ornithischia [14]) appears to be independently derived as all other basal ornithichian taxa show the condition otherwise uniform across basal archosaurs and Theropoda. While speculative, developmental constraints be investigated explain the similarities between ankylosaurs [14] and archosaurian outgroups (i.e., two sets of ceratobranchials).

### Inference of a shift in larynx position

When avian suspension of the larynx caudal to a midline element (or elements) by a new intrinsic muscle, M. cricohyoideus, arose is unclear and depends upon the estimated potential correlate of this transition. Correlates of this shift may include a rostral position of the basihyal or its morphology and degree of ossification (Fig 8). As noted above, a progressive rostral shift in the position of the ceratobranchial-basihyal contact appears to be gradual across Dinosauria and may be independently present within Pterosauria (Fig 8). We propose this trait may have minimally been present in common ancestor of the ornithurine *Hongshanornis* and Aves, when an osseous, narrow midline element is consistently present (Fig 8). However, it may have arisen earlier in Paraves, at the common ancestor of *Microraptor* and Aves and even be implied as arising convergently in pterosaurs, where the ceratobranchials may fuse on the midline (Fig 6B and 6C). The functional significance of a shift in larynx position is also unclear. The only other non-mammalian tetrapods with the larynx caudal to the basihyal are frogs, which produce complex vocalizations with a larynx rather than syrinx-based sound source in birds. While it is possible this novel avian larynx position arose for a respiratory or vocal modulation function [7, 29], it is as likely originally a byproduct of selection on skull shape, tongue function and intraoral food processing that underwent significant transformation within Paraves [46, 48]. Understanding how novelties in locomotor mode, respiration and vocal behavior, and homeothermy may be linked requires a synthetic look at the timing of these core innovations and further fossil data to illuminate their relative timing [46–48].

## Conclusions

Trends in the evolution of tongue structure in archosaurs lie in stark contrast to those observed in lepidosaurs (tuatara, lizards and snakes), taxa that commonly informed early reconstructions of charismatic extinct forms, such as dinosaurs. In lepidosaurs, which show remarkable diversity in hyoid shape, there remains a primary respiratory function for the hyoid elements. The hyobranchial elements (multiple sets of ceratobranchials) show a primarily dorsoventral movement that is deployed during buccal pumping [35]. The hyoid structure shows strong muscular links to the pectoral girdle that are lost in archosaurs [18] and any tongue protrusion is via attached fleshy extensions rather than bony components [1]. In Archosauria, the evolution of novel respiratory mechanisms apparently drove a simplification of the tongue [18] that was retained in most taxa. Only with the evolution of flight (birds and pterosaurs) and in select quadrupedal herbivores was tongue structure elaborated. Shifts in locomotor mode appear to introduce novel selective pressures on food manipulation during feeding and linked changes in diet. While mostly carnivorous non-avian dinosaurs elaborate dentition, extant birds show complex, functionally-linked diversity in rostral shape and tongue structure. Why this appears to be exclusively seen within extant birds (primarily Neognathae) to the exclusion of pterosaurs and other dinosaurs is unexplained. To explore why living birds show such trends, we may need to explore body size dynamics, the diversification of flowering plants or the diversity dynamics of potential prey.

## Supporting information

S1 Fig Major morphological evolution of bony hyoid traits in bird-line archosaurs with new data obtained from Avialae, Palaeognathae, and Neognathae. The hyoid elements are labeled as abbreviation, pg-paraglossal, bh-basihyal, cb-ceratobranchial, ep-epibranchial. Bony hyoid characters include:
(1) origin of the narrow, arrow-shaped basihyal (not always mineralized; see also one specimen of *Microraptor*);
(2) origin of the separate epibranchials and (3) origin of the urohyal;
(4) elongation of the epibranchial, and (5) the paraglossal;
(6) cartilaginous paraglossal, (7) basihyal and paraglossal are connected by soft tissues, (8) basihyal and urohyal not separate;
(9) ossified paraglossal and (10) with mobile connection with the basihyal.
(TIF)

S2 Fig Parsimony-based ancestral state reconstruction of hyoid features summarized in Fig 3 and the supplemental tables. Characters numbers correspond to states described in Supplemental Data Files 1, 2: the character descriptions and matrix.
(JPG)

S3 Fig Skulls of basal archosaurs and non-avian dinosaurs with associated hyoids evaluated. a, *Euparkeria capensis* (SAM 5867); b, *Jeholosaurus shangyuanensis* (IVPP V12530); c, *Gongbusaurus wucaiwanensis*. (IVPP 14559); d, *Massospondylus carinatus* (cast, BP/1/4934); e, *Syntarsus kayentakatae* (MNA V2623); f, *Similicaudipteryx yixianensis* (STM22-6); g, *Sinosauropteryx prima* (NIGP V127586); h, *Linheraptor exquisitus* (cast, IVPP V16923). The ceratobranchials are indicated by the white arrow.
(TIF)

S4 Fig Avialan skulls with associated hyoid elements preserved. a, *Confuciusornis sanctus* (IVPP 13175); b, *Confuciusornis* sp. (STM 13–6); c, *Rapaxavis pani* (DNHM D2522); d, *Sulcavis geeorum* (BMNH ph 000805); e, *Longusunguis kurochkini* (IVPP V17864); f, *Yanornis martini* (IVPP V12558); g and h, photograph and line drawing of *Hongshanornis* sp. (STM 7–56). The hyoid elements are indicated by arrows. Abbreviation: bh, basihyal; cb, ceratobranchial; ep, epibranchial.
(TIF)

S5 Fig The rostral extent of ceratobranchial-basihyal articulation relative to the orbit position in birds and outgroup lepidosaurs (see Fig 1 for *Alligator*). a, *Acanthisitta chloris*; b, *Gambelia wislizenii* (UC MVZ 172830; courtesy of J.A. Masaino); c, *Hongshanornis* sp. (STM 7–56); d, *Hongshanornis longicresta* (latex peel of IVPP V14533); e and f, *Confuciusornis* sp. (IVPP V11548, STM 13–6). The red lines are aligned to the ceratobranchial-basihyal articulation.
(TIF)

S1 Table Material of extinct taxa examined. Published specimens are indicated with an associated reference.
(DOCX)

S2 Table Homologous muscles proposed across reptilians and examined in this project. Muscles experiencing major shifts, or considered as neomorphs of birds, are indicated in bold face; dash lines indicate it is not present. All proposed homologies were reviewed from previous studies and new proposed homologies are indicated with an asterisk (*).
(DOCX)

S1 File Character list used to reconstruct the major transitions of hyolingual evolution with Archosaria. (DOCX)

S2 File Data matrix (character list and coding). (DOCX)
