# Supplementary figures and images for: Convergent evolution of a mobile bony tongue in flighted dinosaurs and pterosaurs
Source: PLoS One. 2018 Jun 20;13(6):e0198078. doi: 10.1371/journal.pone.0198078 (PMC6010247; doi:10.1371/journal.pone.0198078)

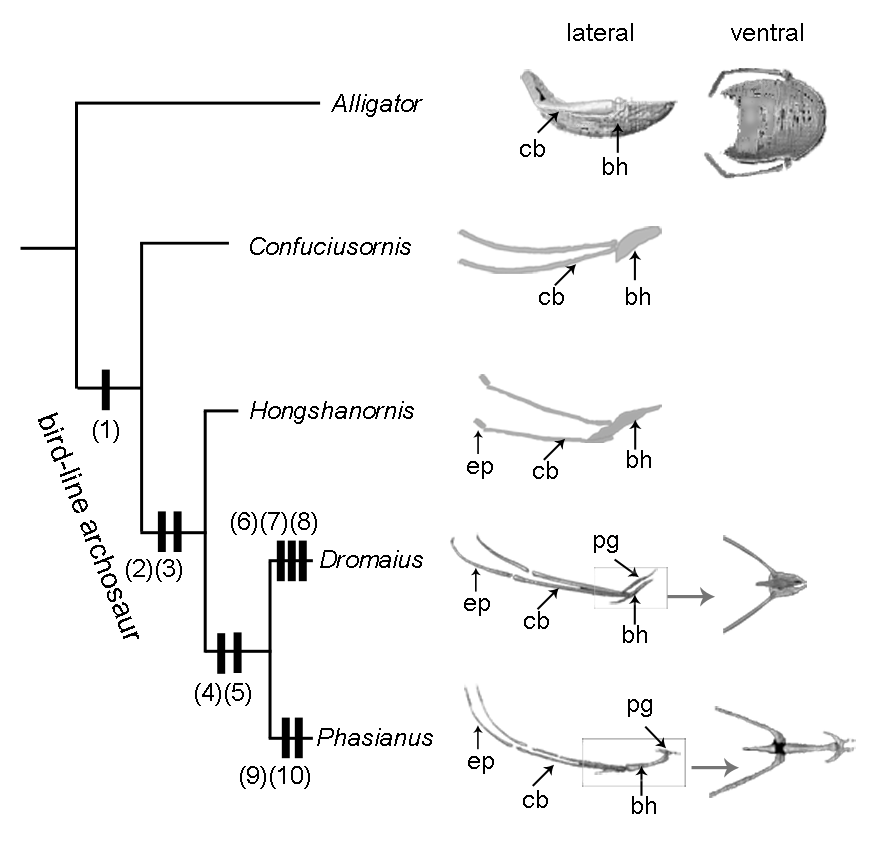

Supplement: S1 Fig — The hyoid elements are labeled as abbreviation, pg-paraglossal, bh-basihyal, cb-ceratobranchial, ep-epibranchial. Bony hyoid characters include: (1) origin of the narrow, arrow-shaped basihyal (not always mineralized; see also one specimen of Microraptor); (2) origin of the separate epibranchials and (3) origin of the urohyal; (4) elongation of the epibranchial, and (5) the paraglossal; (6) cartilaginous paraglossal, (7) basihyal and paraglossal are connected by soft tissues, (8) basihyal and urohyal not separate; (9) ossified paraglossal and (10) with mobile connection with the basihyal. (TIF) [file pone.0198078.s001.tif]

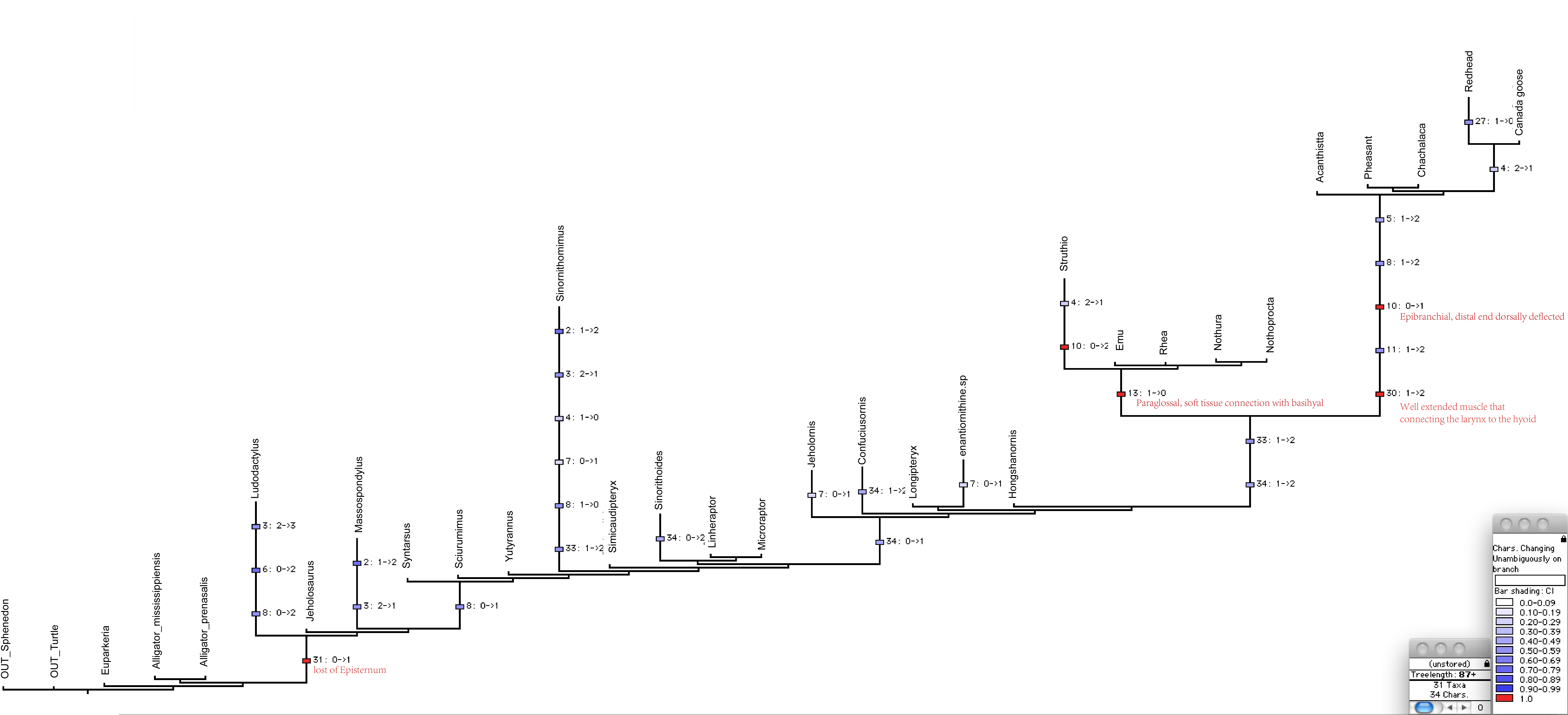

Supplement: S2 Fig — Characters numbers correspond to states described in Supplemental Data Files 1, 2: the character descriptions and matrix. (JPG) [file pone.0198078.s002.jpg]

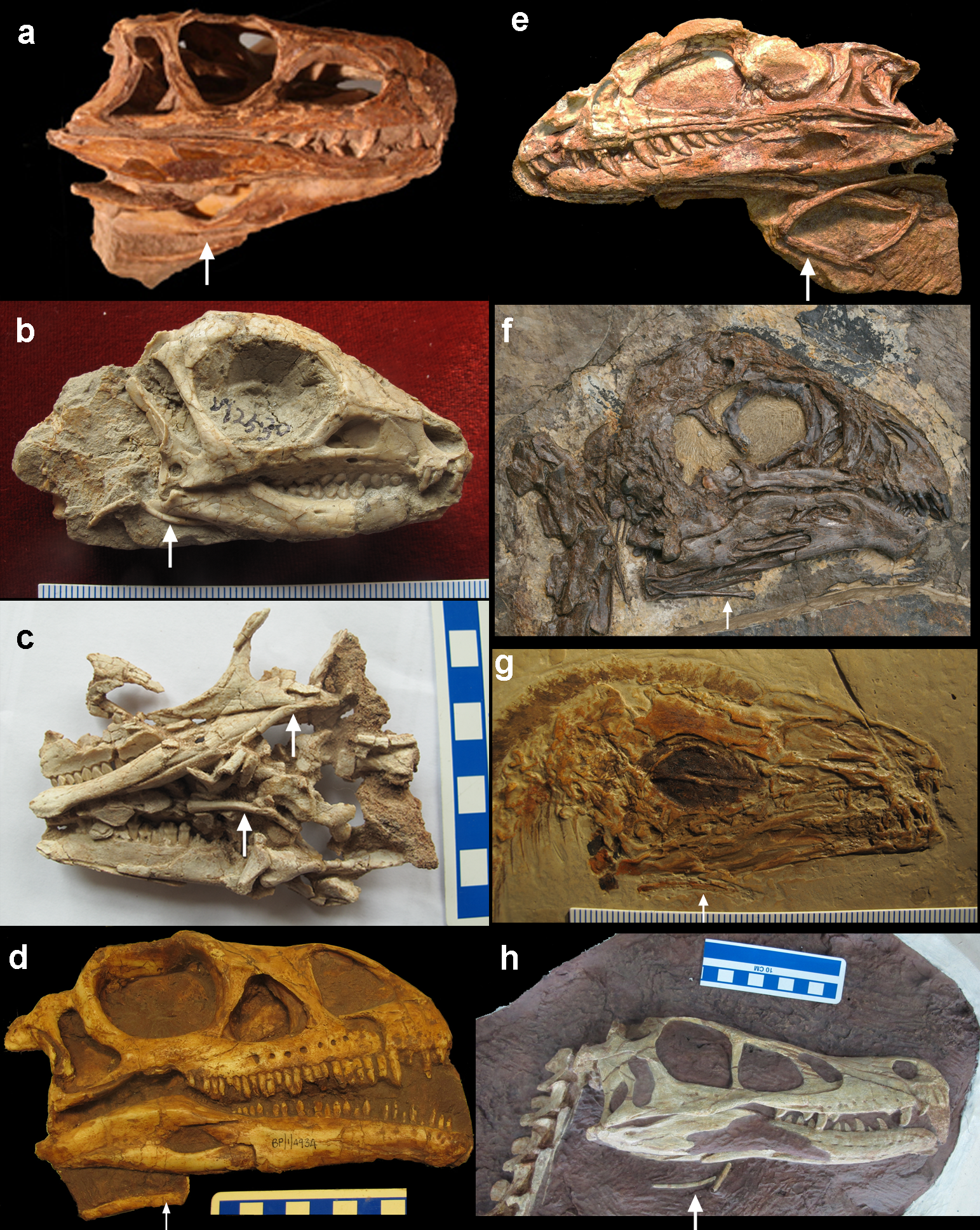

Supplement: S3 Fig — a, Euparkeria capensis (SAM 5867); b, Jeholosaurus shangyuanensis (IVPP V12530); c, Gongbusaurus wucaiwanensis. (IVPP 14559); d, Massospondylus carinatus (cast, BP/1/4934); e, Syntarsus kayentakatae (MNA V2623); f, Similicaudipteryx yixianensis (STM22-6); g, Sinosauropteryx prima (NIGP V127586); h, Linheraptor exquisitus (cast, IVPP V16923). The ceratobranchials are indicated by the white arrow. (TIF) [file pone.0198078.s003.tif]

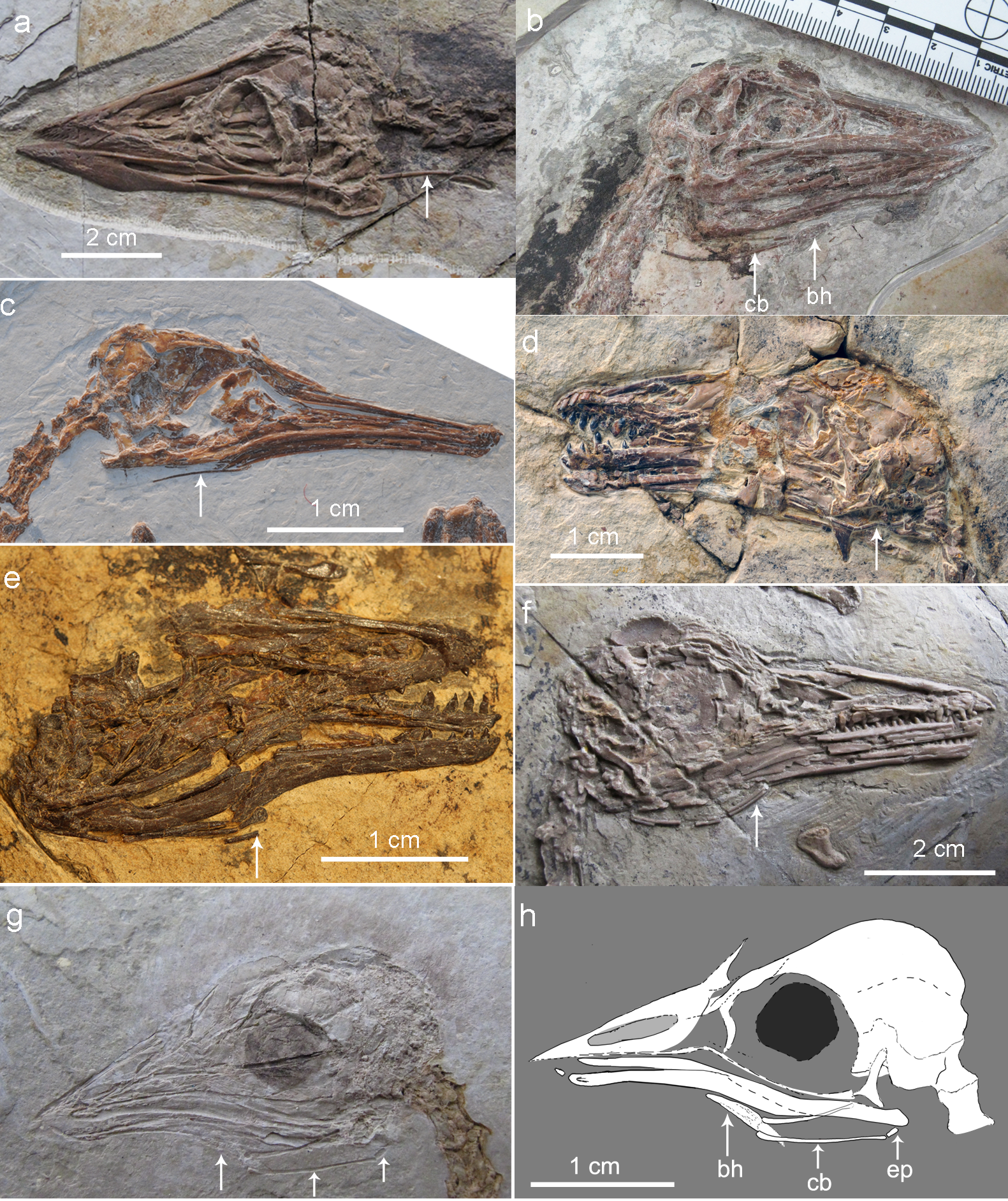

Supplement: S4 Fig — a, Confuciusornis sanctus (IVPP 13175); b, Confuciusornis sp. (STM 13–6); c, Rapaxavis pani (DNHM D2522); d, Sulcavis geeorum (BMNH ph 000805); e, Longusunguis kurochkini (IVPP V17864); f, Yanornis martini (IVPP V12558); g and h, photograph and line drawing of Hongshanornis sp. (STM 7–56). The hyoid elements are indicated by arrows. Abbreviation: bh, basihyal; cb, ceratobranchial; ep, epibranchial. (TIF) [file pone.0198078.s004.tif]
