# Supplementary material for: Convergent evolution of a mobile bony tongue in flighted dinosaurs and pterosaurs
Source: PLoS One. 2018 Jun 20;13(6):e0198078. doi: 10.1371/journal.pone.0198078 (PMC6010247; doi:10.1371/journal.pone.0198078)
Supplement: S1 Table — Published specimens are indicated with an associated reference. (DOCX) [file pone.0198078.s006.docx]

**Supporting Tables**

Table S1 **Material of extinct taxa examined; published specimens are indicated with an associated reference.**

| **Species** | **Number** | **Reference** | **Institution** |
| --- | --- | --- | --- |
| *Euparkeria Capensis* | SAM 5867 | Ewer 1965 | South African Museum |
| *Liaoxipterus brachycephalus* | CAR-0018 | Lü 2015 | Jilin University |
| *Ludodactylus sibbicki* | SMNK PAL 3828 | Frey et al. 2003 | Staatliches Museum Für Naturkunde Karlsruhe |
| *Alligator prenasalis* | SDSM 243 |  | South Dakota School of Mines and Technology |
| *Agilisaurus louderbacki* | ZDM 6011 | Peng 1990 | Zigong Dinosaur Museum, Zigong, China |
| *Jeholosaurus shangyuanensis* | IVPP V12530  IVPP V15719 | Han et al. 2012 | Institute of Vertebrate Palaeontology and Palaeoanthropology, Beijing, China |
| *Gongbusaurus wucaiwanensis* | IVPP 14559 |  | Institute of Vertebrate Palaeontology and Palaeoanthropology, Beijing, China |
| *Jinzhousaurus yangi* | IVPP V12691 | Wang and Xu 2001 | Institute of Vertebrate Palaeontology and Palaeoanthropology, Beijing, China |
| *Gigantspinosaurus sichuanensis* | ZDM 0019 |  | Zigong Dinosaur Museum, Zigong, China |
| *Jiangjunosaurus junggarensis* | IVPP V14724 | Jia et al. 2007 | Institute of Vertebrate Palaeontology and Palaeoanthropology, Beijing, China |
| *Psittacosaurus ordosensis* | IVPP V070888-1 |  | Institute of Vertebrate Palaeontology and Palaeoanthropology, Beijing, China |
| *Yunnanosaurus sp.* | ZLJ 0110 |  | Lufeng World Dinosaur Valley Park, Yunnan |
| *Massospondylus carinatus* | BP/1/4934 | TMM-cast | Vertebrate Paleontology Laboratory, University of Texas at Austin |
| *Syntarsus kayentakatae* | MNA V2623 | Rowe 1989 | Museum of Northern Arizona |
| *Sciurumimus albersdoerferi* | BMMS BK 11 | Rauhut et al. 2012 | Bürgermeister Müller Museum Solnhofen (BMMS) |
| *Carnotaurus sastrei* | MACN-CH 894 | Bonaparte et al. 1990 | Meseo Argentino de Ciencias Naturales |
| *Limusaurus inextricabilis* | IVPP V15923 | Xu et al. 2009a | Institute of Vertebrate Palaeontology and Palaeoanthropology, Beijing, China |
| *Sinosauropteryx prima* | NIGP V127586,  IVPP V12415 | Lingham-Soliar et al. 2007 | Institute of Vertebrate Palaeontology and Palaeoanthropology, Beijing  Nanjing Institute of Geology and Palaeontology, Nanjing |
| *Beipiaosaurus inexpectus* | STM 31-1 | Zheng 2009 | Shandong Tianyu Museum of Nature, Pingyi, Shandong, China |
| *Yutyrannus huali* | ELDM V1001 | Xu et al. 2012 | Erlianhaote Dinosaur Museum, Inner Mongolia |
| *Sinornithomimus dongi* | IVPP 11797-10 | Kobayashi and Lü 2003 | Institute of Vertebrate Palaeontology and Palaeoanthropology, Beijing, China |
| *Similicaudipteryx yixianensis* | STM 22-6 | Xu et al. 2010a | Shandong Tianyu Museum of Nature, Pingyi, Shandong , China |
| *Sinornithoides youngi* | IVPP V9612 | Russell and Dong 1993 | Institute of Vertebrate Palaeontology and Palaeoanthropology, Beijing, China |
| *Anchiornis huxleyi* | IVPP V16055 | Xu et al. 2009b | Institute of Vertebrate Palaeontology and Palaeoanthropology, Beijing, China |
| *Microraptor gui* | IVPP V13320 | Xu et al 2003 | Institute of Vertebrate Palaeontology and Palaeoanthropology, Beijing, China |
| *Microraptor zhaoianus* | BMNHC PH881  And another uncatalogued specimen | Li et al. 2012 | Beijing Museum of Natural History, Beijing, China |
| *Linheroptor exquisitus* | IVPP V 16923 | Xu et al. 2010b | Institute of Vertebrate Palaeontology and Palaeoanthropology, Beijing |
| *Jeholornis prima* | IVPP V13278 | Zhou and Zhang 2002 | Institute of Vertebrate Palaeontology and Palaeoanthropology, Beijing |
| *Jeholornis prima* | BMNHC-PH780 | Meng and Chiappe 2016 | Beijing Museum of Natural History, Beijing, China |
| *Confuciusornis sanctus* | IVPP V13175 |  | Institute of Vertebrate Palaeontology and Palaeoanthropology, Beijing |
| *Confuciusornis sp.* | IVPP V11548 |  | Institute of Vertebrate Palaeontology and Palaeoanthropology, Beijing |
| *Confuciusornis sp.* | STM 13-6 |  | Shandong Tianyu Museum of Nature, Pingyi, Shandong, China |
| *Enantiornithine sp.* | IVPP V13266 |  | Institute of Vertebrate Palaeontology and Palaeoanthropology, Beijing, China |
| *Enantiornithine sp.* | BMNHC-PH877 | Meng and Chiappe 2016 | Beijing Museum of Natural History, Beijing, China |
| *Sapeornis chaoyangensis* | BMNHC-PH1067 | Meng and Chiappe 2016 | Beijing Museum of Natural History, Beijing, China |
| *Protopteryx fengningensis* | BMNHC-PH1060A | Meng and Chiappe 2016 | Beijing Museum of Natural History, Beijing, China |
| *Zhouornis hani* | *BMNHC Ph 756.* | Meng and Chiappe 2016 | Beijing Museum of Natural History, Beijing, China |
| *Sulcavis geeorum* | BMNHC-PH805 | Meng and Chiappe 2016 | Beijing Museum of Natural History, Beijing, China |
| Bohaiornithidae | BMNHC-PH1204 | Meng and Chiappe 2016 | Beijing Museum of Natural History, Beijing, China |
| *Longusunguis kurochkini* | IVPP V17864 | Wang et al. 2014 | Institute of Vertebrate Palaeontology and Palaeoanthropology, Beijing, China |
| *Longipteryx sp.* | STM 9-8 | Zheng 2009 | Shandong Tianyu Museum of Nature, Pingyi, Shandong, China |
| *Rapaxavis pani* | DNHM D2522 | O’Connor et al. 2011 | Dalian Natural History Museum, Dalian, China |
| *Yanornis martini* | IVPP V12558 | Zhou and Zhang 2001 | Institute of Vertebrate Palaeontology and Palaeoanthropology, Beijing, China |
| *Yanornis martini* | BMNHC-PH1043 | Meng and Chiappe 2016 | Beijing Museum of Natural History, Beijing, China |
| *Yanornis martini* | BMNHC-PH928 | Meng and Chiappe 2016 | Beijing Museum of Natural History, Beijing, China |
| *Yanornis martini* | XHPM-1205 | Meng and Chiappe 2016 | Xinghai Museum of Prehistoric Life of Dalian, Dalian, China |
| *Yanornis sp.* | STM 8-117 | Zheng 2009 | Shandong Tianyu Museum of Nature, Pingyi, Shandong, China |
| *Hongshanornis longicresta* | IVPP V14533 | Zhou and Zhang 2005 | Institute of Vertebrate Palaeontology and Palaeoanthropology, Beijing, China |
| *Hongshanornis longicresta* | DNHM D2945 | Chiappe et al. 2014 | Dalian Natural History Museum, Dalian, China |
| *Hongshanornis sp.* | STM 7-56 | Zheng 2009 | Shandong Tianyu Museum of Nature, Pingyi, Shandong, China |
| *Longicrusavis houi* | PKUP-V1069 | O'Connor et al. 2010 | Peking University Paleontological Collections, Beijing, China |
| *Gansus zheni* | BMNHC-PH1343 | Meng and Chiappe 2016 | Beijing Museum of Natural History, Beijing, China |
