# Supplementary material for: Convergent evolution of a mobile bony tongue in flighted dinosaurs and pterosaurs
Source: PLoS One. 2018 Jun 20;13(6):e0198078. doi: 10.1371/journal.pone.0198078 (PMC6010247; doi:10.1371/journal.pone.0198078)
Supplement: S2 Table — Muscles experiencing major shifts, or considered as neomorphs of birds, are indicated in bold face; dash lines indicate it is not present. All proposed homologies were reviewed from previous studies and new proposed homologies are indicated with an asterisk (*). (DOCX) [file pone.0198078.s007.docx]

**Supporting Tables**

Table S2. **Homologous muscles proposed across reptilians and examined in this project; muscles experiencing major shifts, or considered as neomorphs of birds, are indicated in bold face; dash lines indicate it is not present. All proposed homologies were reviewed from previous studies and new proposed homologies are indicated with an asterisk (*).**

| Taxa/  Muscle group | Turtle  Schumacher 1973 | *Sphenodon*  Rieppel  1977 | Crocodylia  (this paper) | Paleognathae  (this paper) | Neognathae  (this paper) |
| --- | --- | --- | --- | --- | --- |
| Mandibular muscles | M. intermandibularis | M. intermandibularis | M. intermandibularis | M. intermandibularis | M. intermandibularis |
|  | M. constrictor colli | M. constrictor colli | M. constrictor colli | M. constrictor colli  (weakly developed) | M. constrictor colli (weakly developed) |
| Hyobranchial muscles  or suprahyoid muscles | M. branchiomandibularis visceralis and M. branchiohyoideus | M. branchiohyoideus, or M. ceratohyoideus | M. branchiomandibularis visceralis | **M. branchiomandibularis (only one head)** | **M. branchiomandibularis one or two heads (*)** |
|  | ----- | ----- | ----- | **M. interceratobranchialis** | **M. interceratobranchialis** |
|  | ----- | ----- | ----- | **M. serpihyoideus** | **M. serpihyoideus and M. stylohyoideus** |
| Hypobranchial muscles (rostral)  or glossal muscles | M. geniohyoideus and M. genioglossus | M. geniohyoideus and M. genioglossus | M. geniohyoideus  and M. genioglossus | M. geniohyoideus | (atrophies through development) |
|  | M. hyoglossus | M. hyoglossus | M. hyoglossus | M. ceratoglossus | M. ceratoglossus **(*)** |
|  | ----- | ----- | ----- | **M. hypoglossus (obliques)** | **M. hypoglossus (cranialis and obliques)** |
|  | ----- | ----- | M.cricoarytenoid muscle | **M. cricohyoideus** | **M. cricohyoideus(*)** |
|  | ? | M. mandibulohyoideus (*) | M. branchiomandibularis spinalis | ----- | ----- |
| Hypobranchial muscles (caudal) | M. episternohyoideus | M. sternohyoideus | M. episternobranchialis and M. episternotendineous | **M. sternoltrachealis and M. tracheolateralis** | **M. sternoltrachealis and M. tracheolateralis** |
|  | M. omohyoideus | M. coracohyoideus | M. coracohyoideus | **M. cleidohyoideus or M. cleidotrachealis** | **M. cleidohyoideus or M. cleidotrachealis** |
