# Supplementary material for: Convergent evolution of a mobile bony tongue in flighted dinosaurs and pterosaurs
Source: PLoS One. 2018 Jun 20;13(6):e0198078. doi: 10.1371/journal.pone.0198078 (PMC6010247; doi:10.1371/journal.pone.0198078)
Supplement: S1 File — (DOCX) [file pone.0198078.s008.docx]

**Supporting data**

**Character list and optimizations**

**Supplemental Data File 1. Character list used to reconstruct the major transitions of hyolingual evolution with Archosaria.**

Character descriptions and notes:

*A: Skeletal hyoid elements*

1. Ceratohyal: (0) present; (1) absent.

Note: Ceratohyal is only present in outgroup taxa (i.e., Lepidosaurs).

1. Ceratobranchial II (CB II, second pair of the ceratobranchials): (0) present; (1) absent.

Note: CBII is present in outgroups, e.g., lepidosaurs and turtles; based on current evidence, loss of CB II is proposed synapomorphy for Archosauria (Li and Clarke 2015).

1. Basihyal (middle-line element), ossification: (0) well ossified; (1) moderately ossified; (2) cartilaginous; (3) absent.

Note: observations were made based on a large number of specimens from museum collections (USNM and NMNH collections) for birds; it is well ossified in Neognathae, moderately ossified in Palaeognathae; while, ossification is lacked in most non-avian dinosaurian taxa, except for a few avialans (e.g., *Confuciusornis* and *Hongshanornis*).

1. Ceratobranchial I (CB I, first pair of ceratobranchials): (0) robust; (1) moderate; (2) slim.
2. Ceratobranchial I, articulation with the midline basihyal: (0) absent; (1) a weakly developed knob; (2) well-developed knob present.

Note: An avian neomorph, the well-developed knob is present in Neognathae, moderately developed in Palaeognathae; *Jeholornis*, numerous Enantiornithes and Ornithurines show the derived condition for this feature.

1. CB I, space between rostral tips: (0) large, CB I are widely separated; (1) small, close to each other. (2) contacting each other.
2. CB I, general shape: (0) straight or slightly dorsally deflected; (1) strongly dorsally deflected.

Note: Only state one in *Jeholornis*, several enantiornithine specimens (e.g., IVPP V13266), and outgroup taxa, the feature were scored as 1; this feature appears to be convergent on the strongly deflected epibranchial in Neognathae.

1. CB I, cranial extension in lateral view: (0) caudal to, or close to the middle, of the orbit; (1) rostrally bypass the middle of orbit; (2) significantly rostrally well beyond orbit.
2. Epibranchial: (0) absent; (1) short; (2) elongate. (ordered)

Note: we consider these states to represent a morphocline. A transition in length is observed between basal ornithurines and Neognathae.

1. Epibranchial, distal end: (0) slightly dorsally deflected; (1) strongly dorsally deflected; (2) ventrally deflected. State 2 is derived in some Palaeognathae
2. Epibranchial, ossification: (0) cartilaginous; (1) weakly ossified; (2) well ossified.
3. Paraglossal: (0) absent; (1) present.

Note: currently paraglossal is only found in Aves within Archosauria and was reconstructed as an synapomorphy for Aves.

1. Paraglossal, connection with basihyal: (0) soft tissue connection; (1) direct connection with well-developed joint.

*B: Hyoid related muscles*

1. *M. branchiohyoideus*, or *M. ceratohyoideus* (in lepidosaurs) or the proposed homologous muscles (M. branchiomandibularis[visceral]), origin on hyoid: (0) CB I and Ceratohyal; (1) only CB I; (2) both the CB I and epibranchial, but mainly on the epibranchial.
2. M. branchiomandibularis (visceral) or the homologous muscles: (0) one head; (1) two heads. (See Fig 9: It has only one hear in palaeognathous birds (*Rhea americana* and *Dromaius novaehollandiae*; Fig 9A, B); it is weakly differentiated in Tinamou (e.g., *Nothura maculosa*) and is well distinctive in having two heads in *Megapodius pritchardii* and *Phasianus colchicus* (Fig 9: C, D, E); the rostral portion extends further craniad in Neognathae than that in Palaeognathae and *Alligator* (Fig 9: F).

Note: M. branchiomandibularis is adopted from Avian anatomical terminology (Vanden Berge and Zweers 1993), for the scoring of muscular feature in lepidosaurs, we scored the condition for the potential homologous M. branchiohyoideus, or M. ceratohyoideus muscle instead. The same is true for character 16.

1. M. branchiomandibularis (visceral), rostrocaudal extent: (0) short; (1) medium; (2) long.

Note: The elongated epibranchial in Neognathae is proposed to be a direct skeletal correlate of the rostrocaudal extension of the M. branchiomandibularis.

1. M. branchimmadibularis (visceral) or homologous muscles, position to the M. intermandibularis: (0) one head, outside (ventral to) the M. intermandibularis; (1) one head outside, another inside; (2) only one head, inside (Huang *et al*. 1999).
2. M. intermandibularis cranialis, development: (0) well developed and cover the ventral side of the buccal floor; (1) moderately developed; (2) weakly developed or absent, very thin.

Notes: M. intermandibularis is well developed in *Rhea americana*, *Nothura maculosa*, *Dromaius novaehollandiae*, *Struthio camelus*, and *Alligator* *mississippiensis* (See Fig 10: A-E), but not in *Phasianus colchicus* (F); it is also relatively thinner in birds than in *Alligator*.

1. M. branchiomandibularis spinalis: (0) present; (1) absent.

Note: M. branchiomandibularis spinalis is distinctive from M. branchiomadibularis (visceral) by its innervation by the hypoglossal nerve (CN XII) rather than the glossopharyngeal nerve (CN IX) (Schumacher 1973). It is only reported from *Alligator*. In addition, the topography and morphology of the muscle are highly similar to other hypobranchial muscles (based on data from *Alligator*). Here we considered the homologous muscles in the outgroups would be M. mandibulohyoideus.

1. Separated M. coracohyoideus (M. omohyoideus) or homologous muscles: (0) present; (1) absent.
2. M. coracohyoideus, or proposed homologous muscle (e.g. M. omohyoideus in *Alligator*; the M. cleidohyoideus and the M. cleidotrachealis in birds), origin: (0) interclavicles, clavicle (or the homologous episternum); (1) coracoid; (2) furcula.

Note: The furcula apophysis and the cranial-medial border of the furcula are reported, and confirmed, as the insertion site for the muscles in several birds (e.g., *Phasianus*, *Chen* and *Athya*). Therefore, skeletal evidence of furcula was considered as a potential correlation for tracing the muscular changes among extinct archosaurs. Fig 8 (A, B: *Alligator* and C, D: *Aythya*).

1. M. coracohyoideus, or potential homologous muscle, insertion: (0) hyoid element and tracheal rings; (1) only on the trachea and the larynx.
2. M. sternohyoideus or homologous muscles (e.g. episternobranchiotendineus and/or episternobranchialis in *Alligator*; tracheolaryngealis in birds), origin: (0) interclavicle and/or episternum; (1) particularly on the craniolateral region or the craniolateral process of the sternum.

Note: This skeletal correlation was used to estimate the related muscular changes in non-avian dinosaurs as well as avialans. (Theses episterno-hyoid muscle complex are generally not well developed in birds. They reduced their attachment from hyoids to the larynx and the tracheal. See Fig 11, the well-developed robust episterno-hyoid complex in alligator (A, B); the various slim muscles proposed to be homologous in birds including the M. sternotrachealis (c, d: *Chen caerulescens*), tracheolateralis (E, F: *Aythya americana* and *Rhea americana*) as well as the M. cricohyoides (G, H, I: *Nothura maculosa*, *Megapodius pritchardii*, and *Aythya americana*). The condition in alligator probably represents the ancestral condition for Archosauria.

1. M. sternohyoideus or homologous muscles, insertion: (0) hyoid elements and/or trachea; (1) only on the trachea and/or the larynx.
2. Reptilian hyoglossal or homologous muscles (i.e., M. ceratoglossal in birds): (0) well developed; (1) slim.
3. M. geniohyoideus or M. genioglossus: (0) well developed; (1) weakly developed (relatively thin); (2) absent. (M. genioglossus and/or geniohyoideus are well developed in *Dromaius novaehollandiae*, *Nothura maculosa*, *Rhea americana*, *Struthio camelus*, as well as in *Alligator*; but totally reduced in most Neognathae dissected (*Phasianus colchicus*, *Aythya americana*); compared to *Alligator*, it is relatively thin and less developed in Palaeognathae).
4. Avian hyoid retractor muscles (M. serpihyoideus and M. stylohyoideus), cranial extension: (0) moderate, only to the urohyal; (1) extend to the basihyal.
5. M. interceratobranchialis: (0) absent; (1) present.

M. interceratobranchialis is a derived muscle that is only found in birds.

*C: Larygeal apparatus*

1. Larynx, position relative to the basihyal: (0) on the basihyal; (1) caudal to the basihyal. (The larynx is above the basihyal in *Alligator*, but caudal to the basihyal in all birds: *Dromaius novaehollandiae*, *Phasianus colchicus*, and *Nothura maculosa* examined; Fig 12: A-D).
2. The muscle that connect the larynx to the hyoid (e.g., M. cricohyoideus): (0) absent; (1) present, short; (2) present, well extended.

Notes: M. cricohyoideus is a derived muscle that is only found in birds, e.g., in *Nothura maculosa*, *Megapodius pritchardii*, and *Aythya americana* (Fig 11: G-I); the muscle was proposed to be homologous to the cranial portion of the M. sternohyoideus in non-avian reptiles (Edgeworth 1935).

*D: Potential skeletal correlates outside the hyoid elements and others*

1. Episternum or interclavicle: (0) present; (1) absent.

Note: Present in extant outgroup as well as the *Euparkeria* (probably ancestral to Archosauria, but lost in dinosaurian evolution)

1. Furcula: (0) absent; (1) present.
2. Cervical vertebrae, number: (0) <8; (1) 9-11; (2) more than 12.
3. Tooth reduction: (0) none; (1) partially edentulous; (2) fully edentulous.

**Supplemental Data File 2. Data matrix (character list and coding)**

| Species/Character | 1 | 2 | 3 | 4 | 5 | 6 | 7 | 8 | 9 | 10 | 11 | 12 | 13 | 14 | 15 | 16 | 17 | 18 | 19 | 20 | 21 | 22 | 23 | 24 | 25 | 26 | 27 | 28 | 29 | 30 | 31 | 32 | 33 | 34 |
| --- | --- | --- | --- | --- | --- | --- | --- | --- | --- | --- | --- | --- | --- | --- | --- | --- | --- | --- | --- | --- | --- | --- | --- | --- | --- | --- | --- | --- | --- | --- | --- | --- | --- | --- |
| *Acanthistta chloris* | 1 | 1 | 0 | 2 | 2 | 1 | 0 | 2 | 2 | 1 | 2 | 1 | 1 | 2 | ? | 2 | ? | 2 | 1 | 0 | ? | 1 | 1 | 1 | 1 | 2 | 1 | 1 | 1 | 2 | 1 | 1 | 2 | 2 |
| *Phasianus colchicus* | 1 | 1 | 0 | 2 | 2 | 1 | 0 | 2 | 2 | 1 | 2 | 1 | 1 | 2 | 1 | 2 | 1 | 2 | 1 | 0 | 2 | 1 | 1 | 1 | 1 | 2 | 1 | 1 | 1 | 2 | 1 | 1 | 2 | 2 |
| *Chachalaca vetula* | 1 | 1 | 0 | 2 | 2 | 1 | 0 | 2 | 2 | 1 | 2 | 1 | 1 | 2 | 1 | 2 | 1 | 2 | 1 | 0 | 2 | 1 | 1 | 1 | 1 | 2 | 1 | 1 | 1 | 2 | 1 | 1 | 2 | 2 |
| *Aythya americana* | 1 | 1 | 0 | 1 | 2 | 1 | 0 | 2 | 2 | 1 | 2 | 1 | 1 | 2 | 0 | 2 | 2 | 2 | 1 | 0 | 2 | 1 | 1 | 1 | 1 | 2 | 0 | 1 | 1 | 2 | 1 | 1 | 2 | 2 |
| *Branta canadensis* | 1 | 1 | 0 | 1 | 2 | 1 | 0 | 2 | 2 | 1 | 2 | 1 | 1 | 2 | 0 | 2 | 2 | 2 | 1 | 0 | 2 | 1 | 1 | 1 | 1 | 2 | 1 | 1 | 1 | 2 | 1 | 1 | 2 | 2 |
| *Dromaius novaehollandiae* | 1 | 1 | 1 | 2 | 1 | 1 | 0 | 1 | 2 | 0 | 1 | 1 | 0 | 2 | 0 | 1 | 0 | 1 | 1 | 0 | ? | 1 | 1 | 1 | 1 | 1 | 0 | 1 | 1 | 1 | 1 | 1 | 2 | 2 |
| *Rhea americana* | 1 | 1 | 1 | 2 | 1 | 1 | 0 | 1 | 2 | 0 | 1 | 1 | 0 | 2 | 0 | 1 | 0 | 1 | 1 | 0 | ? | 1 | 1 | 1 | 1 | 1 | 0 | 1 | 1 | 1 | 1 | 1 | 2 | 2 |
| *Nothura maculosa* | 1 | 1 | 1 | 2 | 1 | 1 | 0 | 1 | 2 | 0 | 2 | 1 | 0 | 2 | 0 | 1 | 0 | 1 | 1 | 0 | ? | 1 | 1 | 1 | 1 | 1 | 0 | 1 | 1 | 1 | 1 | 1 | 2 | 2 |
| *Nothoprocta perdicaria* | 1 | 1 | 1 | 2 | 1 | 1 | 0 | 1 | 2 | 0 | 2 | 1 | 0 | 2 | 0 | 1 | 0 | 1 | 1 | 0 | ? | 1 | 1 | 1 | 1 | 1 | 0 | 1 | 1 | 1 | 1 | 1 | 2 | 2 |
| *Struthio camelus* | 1 | 1 | 1 | 1 | 1 | 1 | 0 | 1 | 2 | 2 | 1 | 1 | 0 | 2 | 0 | 1 | 0 | 1 | 1 | 0 | ? | 1 | 1 | 1 | 1 | 1 | 0 | 1 | 1 | 1 | 1 | 1 | 2 | 2 |
| *Hongshanornis longicresta* | 1 | 1 | 1/2 | 2 | 1 | 1 | 0 | 1 | 1 | 0 | 0/1 | ? | ? | ? | ? | ? | ? | ? | ? | ? | ? | ? | ? | ? | ? | ? | ? | ? | ? | ? | 1 | 1 | 1 | 1 |
| *Longipteryx chaoyangensis* | 1 | 1 | 2 | 2 | 1 | 1 | 0 | 1 | 0 | - | ? | ? | ? | ? | ? | ? | ? | ? | ? | ? | ? | ? | ? | ? | ? | ? | ? | ? | ? | ? | 1 | 1 | 0 | 1 |
| *Enantiornithine sp.* (IVPP V13266). | 1 | 1 | 2 | 2 | 1 | 1 | 1 | 1 | 0 | ? | ? | ? | ? | ? | ? | ? | ? | ? | ? | ? | ? | ? | ? | ? | ? | ? | ? | ? | ? | ? | 1 | 1 | ? | 1 |
| *Confuciusornis sanctus* | 1 | 1 | 1/2 | 2 | 1 | 1 | 0 | 1 | 0 | - | ? | ? | ? | ? | ? | ? | ? | ? | ? | ? | ? | ? | ? | ? | ? | ? | ? | ? | ? | ? | 1 | 1 | 1 | 2 |
| *Jeholornis prima.* | 1 | 1 | 2 | 2 | 2 | 1 | 1 | ? | 0 | - | ? | ? | ? | ? | ? | ? | ? | ? | ? | ? | ? | ? | ? | ? | ? | ? | ? | ? | ? | ? | 1 | 1 | 1 | 1 |
| *Linheraptor exquisitus* | 1 | 1 | 2 | 2 | 0 | ? | ? | 1 | 0 | - | ? | ? | ? | ? | ? | ? | ? | ? | ? | ? | ? | ? | ? | ? | ? | ? | ? | ? | ? | ? | 1 | ? | 1 | 0 |
| *Microraptor gui* | 1 | 1 | 2 | 2 | 0 | ? | 0 | 1 | 0 | - | ? | ? | ? | ? | ? | ? | ? | ? | ? | ? | ? | ? | ? | ? | ? | ? | ? | ? | ? | ? | 1 | 1 | 1 | 0 |
| *Sinornithoides youngi* | 1 | 1 | 2 | 1 | 0 | 1 | 0 | 1 | 0 | - | ? | ? | ? | ? | ? | ? | ? | ? | ? | ? | ? | ? | ? | ? | ? | ? | ? | ? | ? | ? | 1 | ? | ? | 2 |
| *Simicaudipteryx yixianensis* | 1 | 1 | 2 | 1 | 1 | ? | 0 | 1 | 0 | ? | ? | ? | ? | ? | ? | ? | ? | ? | ? | ? | ? | ? | ? | ? | ? | ? | ? | ? | ? | ? | 1 | ? | ? | ? |
| *Sinornithomimus dongi* | 1 | 2 | 1 | 0 | ? | 0 | 1 | 0 | - | ? | ? | ? | ? | ? | ? | ? | ? | ? | ? | ? | ? | ? | ? | ? | ? | ? | ? | ? | ? | 1 | ? | 1 | 2 | ? |
| *Yutyrannus huali* | ? | ? | ? | ? | ? | ? | ? | ? | ? | ? | ? | 1 | 1 | 2 | 1 | 0 | ? | 0 | 1 | 0 | - | ? | ? | ? | ? | ? | ? | ? | ? | ? | ? | ? | ? | ? |
| *Sciurumimus albersdoerferi* | 1 | 1 | 2 | 1 | 0 | ? | 0 | 1 | 0 | - | ? | ? | ? | ? | ? | ? | ? | ? | ? | ? | ? | ? | ? | ? | ? | ? | ? | ? | ? | ? | 1 | 1 | 1 | 0 |
| *Syntarsus kayentakatae* | 1 | 1 | 2 | 1 | 0 | ? | 0 | 1 | 0 | - | ? | ? | ? | ? | ? | ? | ? | ? | ? | ? | ? | ? | ? | ? | ? | ? | ? | ? | ? | ? | ? | 1 | ? | 0 |
| *Massospondylus carinatus* | 1 | 2 | 1 | 0 | 0 | 0 | 1 | 0 | - | ? | ? | ? | ? | ? | ? | ? | ? | ? | ? | ? | ? | ? | ? | ? | ? | ? | ? | ? | ? | ? | ? | 1 | 0 | ? |
| *Jeholosaurus shangyuanensis* | ? | ? | ? | ? | ? | ? | ? | ? | ? | ? | ? | ? | ? | 1 | 1 | 2 | 1 | 0 | 0 | 0 | 1 | 0 | - | ? | ? | ? | ? | ? | ? | ? | ? | ? | ? | ? |
| *Alligator mississippiensis* | 1 | 1 | 2 | 0 | 0 | 0 | 1 | 0 | 0 | - | ? | 0 | ? | 1 | 1 | 0 | 0 | 0 | 0 | 0 | 1 | 0 | 0 | 0 | 0 | 0 | ? | 0 | 0 | 0 | 0 | 0 | 9 | 0 |
| *Alligator prenasalis* | 1 | 1 | 2 | 0 | 0 | 0 | ? | 0 | 0 | - | ? | ? | ? | ? | ? | ? | ? | ? | ? | ? | ? | ? | ? | ? | ? | ? | ? | ? | ? | ? | ? | 0 | ? | 0 |
| *Ludodactylus sibbicki* (Pterosaur) | 1 | 1 | 3 | 2 | 0 | 2 | 0 | 2 | 0 | - | ? | ? | ? | ? | ? | ? | ? | ? | ? | ? | ? | ? | ? | ? | ? | ? | ? | ? | ? | ? | 1 | 0 | 9 | 0 |
| *Euparkeria capensis* | 1 | 1 | 2 | 2 | 0 | ? | 0 | 0 | 0 | - | ? | ? | ? | ? | ? | ? | ? | ? | ? | ? | ? | ? | ? | ? | ? | ? | ? | ? | ? | ? | 0 | 0 | 0 | 0 |
| *OUT Sphenedon punctatus* | 0 | 0 | 0 | 2 | 1 | 1 | 1 | 0 | 0 | - | ? | 0 | ? | 0 | 0 | 0 | 0 | 0 | 0 | 0 | 0 | 0 | 0 | 0 | 0 | 0 | ? | 0 | 0 | 0 | 0 | 0 | 0 | ? |
| *OUT Turtle* | 1 | 0 | 0 | 0 | 1 | 1 | 1 | 0 | 0 | - | ? | 0 | ? | 0 | 1 | 0 | 0 | 0 | 1 | 0 | 1 | 0 | ? | ? | 0 | 0 | ? | 0 | 0 | 0 | 0 | 0 | 0 | 2 |

**Supplemental References**

Chiappe LM et al. 2014. A new specimen of the Early Cretaceous bird *Hongshanornis longicresta*: insights into the aerodynamics and diet of a basal ornithuromorph. *PeerJ*, 2, e234.

Chiappe LM and Meng Q. 2016. Birds of stone. Johns Hopkins University Press.

Ewer RF. 1965. The anatomy of the thecodont reptile *Euparkeria capensis* Broom. *Philos. T. Roy. Soc. B.*, 379–435*.*

Frey ED, Martill M, Buchy M. 2003. A new crested ornithochierid from the Lower Cretaceous of northeastern Brazil and the unusual death of unusual pterosaur. In *Evolution and Palaeobiology of Pterosaurs*, (eds E Buffetaut, and J-M Mazin), Geological Society, London, Special Publication, 217, 55–63.

Han FL, Barrett PM, Butler RJ, Xu X. 2012 Postcranial anatomy of *Jeholosaurus shangyuanensis* (Dinosauria, Ornithischia) from the Lower Cretaceous Yixian Formation of China. *J. Vertebr. Paleontol*. 32, 1370–1395.

Jia C, Foster CA, Xu X, Clark JM. 2007. The first stegosaur (Dinosauria, Ornithischia) from the Upper Jurassic Shishugou Formation of Xinjiang, China. *Acta Geol. Sin. (English Edition)* 81, 351–356.

Kobayashi Y, Lu JC. 2003. A new ornithomimid dinosaur with gregarious habits from the Late Cretaceous of China. *Acta Palaeontol. Pol.* 48, 235–259.

Li Q et al. 2012. Reconstruction of *Microraptor* and the evolution of iridescent plumage. *Science* 335, 1215–1219.

Li Z, Zhou Z, Wang M, Clarke JA. 2014. A new specimen of large-bodied basal enantiornithine *Bohaiornis* from the Early Cretaceous of China and the inference of feeding ecology in Mesozoic birds. *J. Paleontol.* 88, 99–108.

Lingham-Soliar T, Feduccia A and Wang X, 2007. A new Chinese specimen indicates that ‘protofeathers’ in the Early Cretaceous theropod dinosaur *Sinosauropteryx* are degraded collagen fibres. *Proc. Roy. Soc. Lond. B*, 274, 1823–1829.

O’Connor JK et al. 2013. A new enantiornithine from the Yixian Formation with the first recognized avian enamel specialization. *J. Vertebr. Paleontol*. 33, 1–12.

O'Connor JK, Gao KQ, Chiappe LM. 2010. A new ornithuromorph (Aves: Ornithothoraces) bird from the Jehol Group indicative of higher-level diversity. *J. Vertebr. Paleontol.* 30, 311–321.

Peng G. 1990. A new small ornithopod (*Agilisaurus louderbacki* gen. et sp. nov.) from Zigong, China. Newsletter of the Zigong Dinosaur Museum (in Chinese). 2, 19–27.

Russell D, Dong Z. 1993. A nearly complete skeleton of a new troodontid dinosaur from the Early Cretaceous of the Ordos Basin, Inner Mongolia, People's Republic of China*. Can. J. Earth Sci*., 30, 2163–2173.

Rowe T. 1989. A new species of the theropod dinosaur Syntarsus from the Early Jurassic Kayenta Formation of Arizona. *J. Vertebr. Paleontol*. 9, 125–136.

Rauhut OW, Foth C, Tischlinger H, Norell MA. 2012 Exceptionally preserved juvenile megalosauroid theropod dinosaur with filamentous integument from the Late Jurassic of Germany*. Proc. Natl. Acad. Sci. U.S.A*., 109, 11746–11751.

Sereno PC, Rao C. 1992. Early evolution of avian flight and perching: new evidence from the Lower Cretaceous of China. *Science*, 255, 845–848.

Xu X et al. 2003. Four-winged dinosaurs from China. *Nature* 421, 335–340.

Xu X et al. 2009a. A Jurassic ceratosaur from China helps clarify avian digital homologies. *Nature*, 459, 940–944.

Xu X et al. 2009b. A new feathered maniraptoran dinosaur fossil that fills a morphological gap in avian origin. *Chi. Sci. Bull.* 54, 430–435.

Xu X et al. 2012. A gigantic feathered dinosaur from the Lower Cretaceous of China. *Nature*, 484, 92–95.

Xu X, Zheng X, You H. 2010a. Exceptional dinosaur fossils show ontogenetic development of early feathers. *Nature* 464, 1338–1341.

Xu X et al. 2010b. A new dromaeosaurid (Dinosauria: Theropoda) from the Upper Cretaceous Wulansuhai Formation of Inner Mongolia, China. *Zootaxa* 2403, 1–9.

Vanden Berge JC, Zweers GA. 1993. In *Nomina Anatomica Avium*, (eds J Baumel, AS King, JE Breazile, HE Evans) pp. 189–250. London, Academic Press.

Zhou Z, Zhang F. 2002. A long-tailed, seed-eating bird from the Early Cretaceous of China. *Nature* 418, 405–409.

Zhou Z, Zhang F. 2001. Two new ornithurine birds from the Early Cretaceous of western Liaoning, China. *Chin. Sci. Bull.*, 46, 1258–1264.
