# Supplementary material for: Convergent evolution of a mobile bony tongue in flighted dinosaurs and pterosaurs
Source: PLoS One. 2018 Jun 20;13(6):e0198078. doi: 10.1371/journal.pone.0198078 (PMC6010247; doi:10.1371/journal.pone.0198078)
Supplement: S2 File — (DOCX) [file pone.0198078.s009.docx]

**Supporting data**

**Supplemental Data File 2. Data matrix (character list and coding)**

| Species/Character | 1 | 2 | 3 | 4 | 5 | 6 | 7 | 8 | 9 | 10 | 11 | 12 | 13 | 14 | 15 | 16 | 17 | 18 | 19 | 20 | 21 | 22 | 23 | 24 | 25 | 26 | 27 | 28 | 29 | 30 | 31 | 32 | 33 | 34 |
| --- | --- | --- | --- | --- | --- | --- | --- | --- | --- | --- | --- | --- | --- | --- | --- | --- | --- | --- | --- | --- | --- | --- | --- | --- | --- | --- | --- | --- | --- | --- | --- | --- | --- | --- |
| *Acanthistta chloris* | 1 | 1 | 0 | 2 | 2 | 1 | 0 | 2 | 2 | 1 | 2 | 1 | 1 | 2 | ? | 2 | ? | 2 | 1 | 0 | ? | 1 | 1 | 1 | 1 | 2 | 1 | 1 | 1 | 2 | 1 | 1 | 2 | 2 |
| *Phasianus colchicus* | 1 | 1 | 0 | 2 | 2 | 1 | 0 | 2 | 2 | 1 | 2 | 1 | 1 | 2 | 1 | 2 | 1 | 2 | 1 | 0 | 2 | 1 | 1 | 1 | 1 | 2 | 1 | 1 | 1 | 2 | 1 | 1 | 2 | 2 |
| *Chachalaca vetula* | 1 | 1 | 0 | 2 | 2 | 1 | 0 | 2 | 2 | 1 | 2 | 1 | 1 | 2 | 1 | 2 | 1 | 2 | 1 | 0 | 2 | 1 | 1 | 1 | 1 | 2 | 1 | 1 | 1 | 2 | 1 | 1 | 2 | 2 |
| *Aythya americana* | 1 | 1 | 0 | 1 | 2 | 1 | 0 | 2 | 2 | 1 | 2 | 1 | 1 | 2 | 0 | 2 | 2 | 2 | 1 | 0 | 2 | 1 | 1 | 1 | 1 | 2 | 0 | 1 | 1 | 2 | 1 | 1 | 2 | 2 |
| *Branta canadensis* | 1 | 1 | 0 | 1 | 2 | 1 | 0 | 2 | 2 | 1 | 2 | 1 | 1 | 2 | 0 | 2 | 2 | 2 | 1 | 0 | 2 | 1 | 1 | 1 | 1 | 2 | 1 | 1 | 1 | 2 | 1 | 1 | 2 | 2 |
| *Dromaius novaehollandiae* | 1 | 1 | 1 | 2 | 1 | 1 | 0 | 1 | 2 | 0 | 1 | 1 | 0 | 2 | 0 | 1 | 0 | 1 | 1 | 0 | ? | 1 | 1 | 1 | 1 | 1 | 0 | 1 | 1 | 1 | 1 | 1 | 2 | 2 |
| *Rhea americana* | 1 | 1 | 1 | 2 | 1 | 1 | 0 | 1 | 2 | 0 | 1 | 1 | 0 | 2 | 0 | 1 | 0 | 1 | 1 | 0 | ? | 1 | 1 | 1 | 1 | 1 | 0 | 1 | 1 | 1 | 1 | 1 | 2 | 2 |
| *Nothura maculosa* | 1 | 1 | 1 | 2 | 1 | 1 | 0 | 1 | 2 | 0 | 2 | 1 | 0 | 2 | 0 | 1 | 0 | 1 | 1 | 0 | ? | 1 | 1 | 1 | 1 | 1 | 0 | 1 | 1 | 1 | 1 | 1 | 2 | 2 |
| *Nothoprocta perdicaria* | 1 | 1 | 1 | 2 | 1 | 1 | 0 | 1 | 2 | 0 | 2 | 1 | 0 | 2 | 0 | 1 | 0 | 1 | 1 | 0 | ? | 1 | 1 | 1 | 1 | 1 | 0 | 1 | 1 | 1 | 1 | 1 | 2 | 2 |
| *Struthio camelus* | 1 | 1 | 1 | 1 | 1 | 1 | 0 | 1 | 2 | 2 | 1 | 1 | 0 | 2 | 0 | 1 | 0 | 1 | 1 | 0 | ? | 1 | 1 | 1 | 1 | 1 | 0 | 1 | 1 | 1 | 1 | 1 | 2 | 2 |
| *Hongshanornis longicresta* | 1 | 1 | 1/2 | 2 | 1 | 1 | 0 | 1 | 1 | 0 | 0/1 | ? | ? | ? | ? | ? | ? | ? | ? | ? | ? | ? | ? | ? | ? | ? | ? | ? | ? | ? | 1 | 1 | 1 | 1 |
| *Longipteryx chaoyangensis* | 1 | 1 | 2 | 2 | 1 | 1 | 0 | 1 | 0 | - | ? | ? | ? | ? | ? | ? | ? | ? | ? | ? | ? | ? | ? | ? | ? | ? | ? | ? | ? | ? | 1 | 1 | 0 | 1 |
| *Enantiornithine sp.* (IVPP V13266). | 1 | 1 | 2 | 2 | 1 | 1 | 1 | 1 | 0 | ? | ? | ? | ? | ? | ? | ? | ? | ? | ? | ? | ? | ? | ? | ? | ? | ? | ? | ? | ? | ? | 1 | 1 | ? | 1 |
| *Confuciusornis sanctus* | 1 | 1 | 1/2 | 2 | 1 | 1 | 0 | 1 | 0 | - | ? | ? | ? | ? | ? | ? | ? | ? | ? | ? | ? | ? | ? | ? | ? | ? | ? | ? | ? | ? | 1 | 1 | 1 | 2 |
| *Jeholornis prima.* | 1 | 1 | 2 | 2 | 2 | 1 | 1 | ? | 0 | - | ? | ? | ? | ? | ? | ? | ? | ? | ? | ? | ? | ? | ? | ? | ? | ? | ? | ? | ? | ? | 1 | 1 | 1 | 1 |
| *Linheraptor exquisitus* | 1 | 1 | 2 | 2 | 0 | ? | ? | 1 | 0 | - | ? | ? | ? | ? | ? | ? | ? | ? | ? | ? | ? | ? | ? | ? | ? | ? | ? | ? | ? | ? | 1 | ? | 1 | 0 |
| *Microraptor gui* | 1 | 1 | 2 | 2 | 0 | ? | 0 | 1 | 0 | - | ? | ? | ? | ? | ? | ? | ? | ? | ? | ? | ? | ? | ? | ? | ? | ? | ? | ? | ? | ? | 1 | 1 | 1 | 0 |
| *Sinornithoides youngi* | 1 | 1 | 2 | 1 | 0 | 1 | 0 | 1 | 0 | - | ? | ? | ? | ? | ? | ? | ? | ? | ? | ? | ? | ? | ? | ? | ? | ? | ? | ? | ? | ? | 1 | ? | ? | 2 |
| *Simicaudipteryx yixianensis* | 1 | 1 | 2 | 1 | 1 | ? | 0 | 1 | 0 | ? | ? | ? | ? | ? | ? | ? | ? | ? | ? | ? | ? | ? | ? | ? | ? | ? | ? | ? | ? | ? | 1 | ? | ? | ? |
| *Sinornithomimus dongi* | 1 | 2 | 1 | 0 | ? | 0 | 1 | 0 | - | ? | ? | ? | ? | ? | ? | ? | ? | ? | ? | ? | ? | ? | ? | ? | ? | ? | ? | ? | ? | 1 | ? | 1 | 2 | ? |
| *Yutyrannus huali* | ? | ? | ? | ? | ? | ? | ? | ? | ? | ? | ? | 1 | 1 | 2 | 1 | 0 | ? | 0 | 1 | 0 | - | ? | ? | ? | ? | ? | ? | ? | ? | ? | ? | ? | ? | ? |
| *Sciurumimus albersdoerferi* | 1 | 1 | 2 | 1 | 0 | ? | 0 | 1 | 0 | - | ? | ? | ? | ? | ? | ? | ? | ? | ? | ? | ? | ? | ? | ? | ? | ? | ? | ? | ? | ? | 1 | 1 | 1 | 0 |
| *Syntarsus kayentakatae* | 1 | 1 | 2 | 1 | 0 | ? | 0 | 1 | 0 | - | ? | ? | ? | ? | ? | ? | ? | ? | ? | ? | ? | ? | ? | ? | ? | ? | ? | ? | ? | ? | ? | 1 | ? | 0 |
| *Massospondylus carinatus* | 1 | 2 | 1 | 0 | 0 | 0 | 1 | 0 | - | ? | ? | ? | ? | ? | ? | ? | ? | ? | ? | ? | ? | ? | ? | ? | ? | ? | ? | ? | ? | ? | ? | 1 | 0 | ? |
| *Jeholosaurus shangyuanensis* | ? | ? | ? | ? | ? | ? | ? | ? | ? | ? | ? | ? | ? | 1 | 1 | 2 | 1 | 0 | 0 | 0 | 1 | 0 | - | ? | ? | ? | ? | ? | ? | ? | ? | ? | ? | ? |
| *Alligator mississippiensis* | 1 | 1 | 2 | 0 | 0 | 0 | 1 | 0 | 0 | - | ? | 0 | ? | 1 | 1 | 0 | 0 | 0 | 0 | 0 | 1 | 0 | 0 | 0 | 0 | 0 | ? | 0 | 0 | 0 | 0 | 0 | 9 | 0 |
| *Alligator prenasalis* | 1 | 1 | 2 | 0 | 0 | 0 | ? | 0 | 0 | - | ? | ? | ? | ? | ? | ? | ? | ? | ? | ? | ? | ? | ? | ? | ? | ? | ? | ? | ? | ? | ? | 0 | ? | 0 |
| *Ludodactylus sibbicki* (Pterosaur) | 1 | 1 | 3 | 2 | 0 | 2 | 0 | 2 | 0 | - | ? | ? | ? | ? | ? | ? | ? | ? | ? | ? | ? | ? | ? | ? | ? | ? | ? | ? | ? | ? | 1 | 0 | 9 | 0 |
| *Euparkeria capensis* | 1 | 1 | 2 | 2 | 0 | ? | 0 | 0 | 0 | - | ? | ? | ? | ? | ? | ? | ? | ? | ? | ? | ? | ? | ? | ? | ? | ? | ? | ? | ? | ? | 0 | 0 | 0 | 0 |
| *OUT Sphenedon punctatus* | 0 | 0 | 0 | 2 | 1 | 1 | 1 | 0 | 0 | - | ? | 0 | ? | 0 | 0 | 0 | 0 | 0 | 0 | 0 | 0 | 0 | 0 | 0 | 0 | 0 | ? | 0 | 0 | 0 | 0 | 0 | 0 | ? |
| *OUT Turtle* | 1 | 0 | 0 | 0 | 1 | 1 | 1 | 0 | 0 | - | ? | 0 | ? | 0 | 1 | 0 | 0 | 0 | 1 | 0 | 1 | 0 | ? | ? | 0 | 0 | ? | 0 | 0 | 0 | 0 | 0 | 0 | 2 |
